# Pleiotropic effects of extended blockade of CSF1R signaling in adult mice

**DOI:** 10.1189/jlb.2A0114-006R

**Published:** 2014-08

**Authors:** Kristin A. Sauter, Clare Pridans, Anuj Sehgal, Yi Ting Tsai, Barry M. Bradford, Sobia Raza, Lindsey Moffat, Deborah J. Gow, Philippa M. Beard, Neil A. Mabbott, Lee B. Smith, David A. Hume

**Affiliations:** * The Roslin Institute and Royal (Dick) School of Veterinary Studies and; † Medical Research Council Centre for Reproductive Health, The Queen's Medical Research Institute, University of Edinburgh, Scotland, United Kingdom

**Keywords:** macrophage, osteoclast, bone, testis, Paneth, Kupffer

## Abstract

We investigated the role of CSF1R signaling in adult mice using prolonged treatment with anti-CSF1R antibody. Mutation of the CSF1 gene in the *op/op* mouse produces numerous developmental abnormalities. Mutation of the CSF1R has an even more penetrant phenotype, including perinatal lethality, because of the existence of a second ligand, IL-34. These effects on development provide limited insight into functions of CSF1R signaling in adult homeostasis. The carcass weight and weight of several organs (spleen, kidney, and liver) were reduced in the treated mice, but overall body weight gain was increased. Despite the complete loss of Kupffer cells, there was no effect on liver gene expression. The treatment ablated OCL, increased bone density and trabecular volume, and prevented the decline in bone mass seen in female mice with age. The *op/op* mouse has a deficiency in pancreatic β cells and in Paneth cells in the gut wall. Only the latter was reproduced by the antibody treatment and was associated with increased goblet cell number but no change in villus architecture. Male *op/op* mice are infertile as a result of testosterone insufficiency. Anti-CSF1R treatment ablated interstitial macrophages in the testis, but there was no sustained effect on testosterone or LH. The results indicate an ongoing requirement for CSF1R signaling in macrophage and OCL homeostasis but indicate that most effects of CSF1 and CSF1R mutations are due to effects on development.

## Abstract (precis)

Prolonged anti-CSF1R prevents age-dependent bone loss in female mice, without the phenotypic consequences of the CSF1R and CSF1 null mutations.

## Introduction

CSF1 controls the proliferation, differentiation, maturation, and survival of cells of the mononuclear phagocyte system [1–4]. The effects of CSF1 are mediated through the CSF1R, a protein tyrosine kinase receptor. Expression of *Csf1r* mRNA is myeloid-restricted in adult animals, and a *Csf1r*-EGFP reporter gene provides a convenient marker for macrophage-lineage cells in transgenic mice [5]. A natural mutation of the *Csf1* gene in mice (*op/op*) produces a reduction in macrophage numbers in most tissues of the body, accompanied by severe growth retardation, osteopetrosis, and deficiencies in sensory, reproductive, and other endocrine systems [3, 4]. A null mutation of *Csf1r* produces even more penetrant phenotypes, including a significant postnatal mortality [6]. A second ligand for CSF1R, IL-34, provides an explanation for the greater impact of receptor depletion. IL-34 appears to be required for the generation of specific macrophage populations, notably microglia in the brain and epidermal Langerhans cells [7, 8].

CSF1 is not available in saturating concentrations in an adult mouse; the concentration is regulated, in part, by macrophage-mediated clearance in the liver and spleen, providing a relatively simple homeostatic control of macrophage numbers [9]. Treatment of mice with CSF1 causes a massive increase in tissue macrophage numbers throughout the body. Surprisingly, this leads to a rapid increase in the size of the liver and hepatocyte proliferation, suggesting a function for CSF1-dependent macrophages in liver homeostasis (unpublished results). Conversely, continuous CSF1R signaling is required for the maintenance of macrophage populations in adult mice. The administration of a blocking mAb against the CSF1R gradually eliminated resident tissue macrophages from many different organs. The antibody did not prevent monocytopoiesis but apparently, prevented maturation of monocytes in peripheral blood to form the nonclassical Ly6C^−^ population [10, 11]. Consequently, the treatment did not prevent macrophage recruitment into inflammatory sites; indeed, the treatment exacerbated pathology in a model of graft-versus-host disease. These basic findings were subsequently repeated using a different mAb [12]. The relatively slow depletion of tissue macrophages in the treated mice suggests that the major effect of anti-CSF1R is to prevent their replacement, via recruitment or local self-renewal. This view has been confirmed recently in models in which tissue macrophages have been acutely depleted, and replenishment is blocked by anti-CSF1R [13]. The slow turnover of tissue macrophages means that after 3 weeks of treatment used previously, mice had only been fully depleted for a short period, and the consequences may not have become evident. Accordingly, in the current study, we aimed to define the impact of long-term treatment with anti-CSF1R.

## MATERIALS AND METHODS

### Ethics statement

All animal work was reviewed and approved by the Ethical Review Panel at The Roslin Institute and R(D)SVS (Scotland, UK) and conducted under the authority of Home Office Project Licenses 60/3828 and 60/4259.

### In vivo studies

M279 is a rat IgG2b mAb, which blocks CSF1 and IL-34 binding to the CSF1R. In previous studies, the optimal dose of M279 was determined by injecting with increasing doses, 3× weekly for 3 weeks, and serum samples taken and assayed by ELISA for circulating CSF1. A dose of 125–400 μg/injection had a maximal increase in circulating CSF1 [10]. Accordingly, in all of the studies detailed herein, mice were treated with 200 μg rat anti-mouse CSF1R antibody (M279; Amgen, Thousand Oaks, CA, USA) or rat IgG (I4131; Sigma, St. Louis, MO, USA), administered by i.p. injection, 3× weekly for 6 weeks. MacGreen EGFP^+^ and EGFP^−^ on the C57BL/6 background, as well as nontransgenic C57BL/6 male and female mice, were treated, starting at 8–9 weeks of age and weighed on each injection day. Following treatment, the C57BL/6 nontransgenic mice were killed by CO_2_ asphyxiation and peripheral blood immediately collected by cardiac puncture into EDTA tubes (K1230; Teklab, Collinsville, IL, USA). Organs and tissues were collected and weighed, animals were eviscerated, and a carcass weight was measured. Both femurs were collected. Bone marrow was flushed from one femur for cellularity and cell subset analysis. The other femur was fixed overnight in formalin and stored in 70% ethanol.

### Flow cytometry

Bone marrow was flushed from a femur from each animal, mechanically disrupted by pipetting, counted, and diluted to 1 × 10^6^ cells in 100 μl PBS. Bone marrow cells (200 μl, 1×10^6^) were stained in the dark at room temperature for 1 h at 4°C with the following antibodies: allophycocyanin anti-mouse CD115 (CSF1R; Clone AFS98) and PerCP/Cy5.5 anti-mouse Ly-6C (Clone HK1.4; both BioLegend, San Diego, CA, USA). Data were collected on a CyAn ADP analyzer (Beckman Coulter, Brea, CA, USA) and analyzed using FlowJo 7.5.5 flow cytometry analysis software (TreeStar, Ashland, OR, USA) and Minitab 16.1.0 (Minitab, State College, PA, USA).

### ELISA

Blood from EDTA tubes was centrifuged at 1000 *g* at room temperature for 15 min. Plasma was collected and stored at −20°C. IGF1 ELISA was preformed, according to the manufacturer's instructions (AC-18F1; Immunodiagnostic Systems, Boldon, UK).

### Immunohistochemistry

Organs were collected at cull, fixed in 4% PFA, and embedded in paraffin wax. Testis sections for immunohistochemistry were deparaffinized, rehydrated, and antigen-retrieved using a citrate buffer epitope retrieval method [10 pounds/square inch (0.68 atmosphere), 125°C, 30 min in citrate buffer, pH 6.0] before blocking of endogenous peroxidase and nonspecific binding sites. For single-color immunodetection, primary antibodies [anti-Mac-2, Cat. No. CL8942AP, Cedarlane, Burlington, Ontario, Canada; CD163 (M-96), Cat. No. sc-33560, Santa Cruz Biotechnology, Santa Cruz, CA, USA; and CD68, Cat. No. ab955, Abcam, Cambridge, UK] were applied individually in normal horse serum and incubated at 4°C for 24 h, followed by incubation for secondary detection [ImmPRESS reagent kit, Cat. Nos. MP-7401 (rabbit CD163), 7402 (mouse CD68), and 7404 (rat Mac-2); Vector Laboratories, Burlingame, CA, USA] for 1 h. Samples were washed in TBS, and 3,3′-diaminobenzidine detection (ImmPACT peroxidase substrates, Cat. No. SK-4105; Vector Laboratories) was used to resolve sites of immunolocalization, whereas hematoxylin was used as a counterstain. Sections were then mounted for downstream analysis and visualized using an Olympus research microscope AX70 Provis (AxioVision Rel.4.8 software; Scotia, NY, USA).

Bones were decalcified, embedded, and sectioned by the R(D)SVS Clinical Pathology Laboratory (Scotland, UK). Postfixation and MicroCT scan, femurs and tibias were decalcified in 14% EDTA, pH 7.0, for 3 days at room temperature and embedded in paraffin wax. Serial sections, 4 μM-thick, were cut from each block and dried overnight at 37°C before a final drying at 60°C for 25 min. Sections were dewaxed in xylene, rehydrated through ethanol, and washed. TRAP staining, using an acid phosphatase leukocyte (TRAP) kit (387-A; Sigma), was carried out, according to the manufacturer's protocol, except incubation time was increased to 2 h, and a counterstain was not used.

For detection of lysozyme in intestinal crypts, 5μM cryosections from PFA-fixed tissues were permeabilized with 50% methanol for 20 min before immuonostaining with rabbit anti-lysozyme mAb (Abcam) and then Alexa-Flour 594 anti-rabbit IgG (Invitrogen, Paisley, Scotland). Sections were counterstained with Alexa-647 phalloidin (Dako, Ely, UK) and examined using a LSM5 confocal microscope (Zeiss, Welwyn Garden City, UK). For quantification of goblet cells and analysis of general intestine villi morphology, sections were treated with PAS (0.5% pararosaniline, 1% sodium metabisulfite) and counterstained with hematoxylin. For dual-color labeling of pancreas, anti-insulin antibody (Cat. No. ab7842; Abcam) was applied at 4°C for 24 h before secondary detection (biotinylated goat anti-guinea pig IgG antibody, Cat. No. BA-7000; Vector Laboratories). Immunofluorescence was visualized using the TSA Plus Cyanine 3 System, Cat. No. NEL744B001KT (PerkinElmer, Waltham, MA, USA), following the manufacturer's instructions. Anti-pancreatic and duodenal homeobox 1 antibody (Cat. No. ab47267; Abcam) was also applied in normal horse serum, and a secondary detection system [ImmPRESS reagent kit, Cat. No. MP-7401 (rabbit); Vector Laboratories] was used. Finally, Cytogreen was used as counterstain, and the sections were mounted for confocal analysis (LSM 710, ZEN 2011 software; Zeiss).

### Analysis of bone architecture by MicroCT

MicroCT analysis was performed at the left distal femoral using a SkyScan 1172 instrument set (Kontlich, Belgium) at 60 kV and 150 μA at a resolution of 5 μm. The images were reconstructed using the SkyScan NRecon program and analyzed using SkyScan CTAn software. A volume of 200 slices was measured, 100 μm proximal to the primary spongiosa.

### Complete blood-count analysis

Blood was collected into 0.5 ml EDTA tubes (Teklab). Total WBC, RBC, and packed cell volume were measured on the ABX Pentra 60 hematology analyzer. WBC differential counts were performed by making a blood smear and counterstaining with Giemsa stain before counting.

### Electrolytes

Serum electrolytes (sodium and potassium) were measured using an IL ILab 650 analyzer (Instrumentation Laboratory, Bedford, MA, USA), using ion-selective electrodes.

### Testosterone and LH assays

Quantification of circulating hormones was carried out as described previously [14, 15].

### RNA extraction, labeling, and microarray hybridization

Total RNA was extracted using TRIzol reagent (Invitrogen) from liver tissue, homogenized in Lysing Matrix D using a FastPrep Instrument (Q.BIOgene, Irvine, CA, USA). RNA was eluted in water, and its quality was assessed with an 2100 Bioanalyzer (Agilent Technologies, Santa Clara, CA, USA) before being used for labeling. Microarray was performed by Edinburgh Genomics at University of Edinburgh (Scotland, UK). Total RNA (50 ng) was amplified using the Pico SL kit (NuGEN, San Carlos, CA, USA); 2.5 μg of the resulting cDNA was biotin-labeled using the NuGEN Encore labeling kit with the one-half volume protocol. The biotin-labeled product was prepared for hybridization, according to the NuGEN protocol for GeneTitan hybridization, using the GeneTitan Hybridization, Wash, and Stain Kits for whole-transcript array plates (PN 901622; both Affymetrix, Santa Clara, CA, USA). The samples were hybridized to Affymetrix Mouse Gene 1.1 ST array plates, using the appropriate hybridization-wash-scan protocol for this plate and the GeneTitan Hybridization, Wash, and Stain Kits for the reagents (Affymetrix). Image generation and the resulting CEL files for analysis were produced in Affymetrix GeneChip Command Console software, version 3.0.1. Initial quality controls were performed in Expression Console. The obtained Affymetrix CEL files were imported into the Genomics Suite software package, version 6.13.0213 (Gene Expression Omnibus: GSE53573; Partek, St. Louis, MO, USA).

### Statistical analysis

Data on all graphs were analyzed using an unpaired *t*-test or Mann-Whitney nonparametric test, as indicated. Results are presented as the mean and sem. All analyses were performed using GraphPad Prism 5.0 (GraphPad Software, San Diego, CA, USA). *P* < 0.05 was considered significant.

## RESULTS

### The effects of prolonged treatment with anti-CSF1R mAb M279

**Figure 1.**
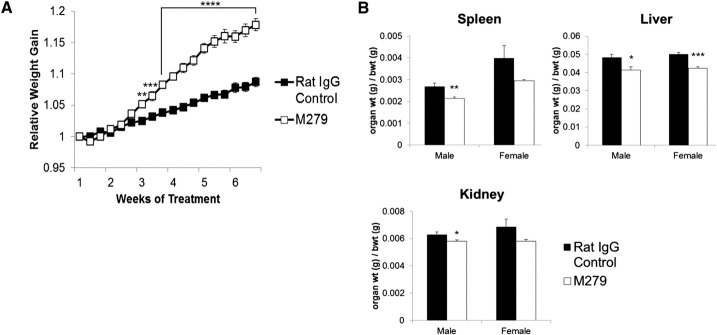
Effect of anti-CSF1R antibody on body and organ weights. Mice were injected with 200 μg rat IgG control or M279, 3× weekly for 6 weeks. Graphs show the mean ± sem. Significance is indicated by *P < 0.05, **P < 0.01, ***P < 0.001, and ****P < 0.0001, using an unpaired *t*-test; *n* = 29 mice/group for body weight (bwt), and *n* = 8 mice/group for organ weights. The groups were assigned to have similar average body weights at the outset. (A) Average relative weight gain (normalized to Day 1) taken before each injection. (B) average spleen:body weight ratio, liver:body weight ratio, and kidney:body weight ratio at the end of treatment. Note that absolute body weight differences between the two groups are <4%, so the reduced organs weights also reflect absolute reductions.

Age- and weight-matched groups of male and female mice were treated with M279, a blocking anti-CSF1R antibody [10], or a rat IgG control, 3× weekly for 6 weeks. CSF1-deficient mice have severe postnatal growth retardation [3], which can be restored by administration of CSF1 [16] and mimicked by anti-CSF1 treatment in the postnatal period [17]. We have shown elsewhere that CSF1-dependent macrophages are a significant source of IGF1 in the postnatal period [18]. After the onset of puberty, the major source of IGF1 for growth is the liver. The adult mice of both sexes continued to gain weight regardless of anti-CSF1R treatment. Indeed, by the beginning of week 3, weight gain in the treated mice showed a clear acceleration relative to controls, and the faster rate of relative weight gain continued throughout the final 4 weeks in the antibody-treated animals (**Fig. 1A**).

The efficacy of the anti-CSF1R treatment was confirmed as a nearly complete reduction of *Csf1r*-EGFP-positive macrophages in the spleen and intestine (Supplemental Fig. 1), extending the findings from the shorter, 3-week treatment [10]. The extended treatment did not produce any more extensive depletion in sites, such as lung and uterus, in which macrophage populations appeared independent of CSF1R signaling [10]. Some additional organs have been analyzed in detail below. In one of the experimental series, we examined an additional group of mice, 3 weeks after cessation of their 6-week anti-CSF1R treatment. Surprisingly, the *Csf1r-*EGFP-positive macrophage numbers in these mice showed no evidence of recovery, although the differential growth rate slowed (Supplemental Fig. 1). However, we do not have an independent measure of the clearance of the antibody in these mice, so we do not know whether CSF1R signaling was restored.

**Figure 2.**
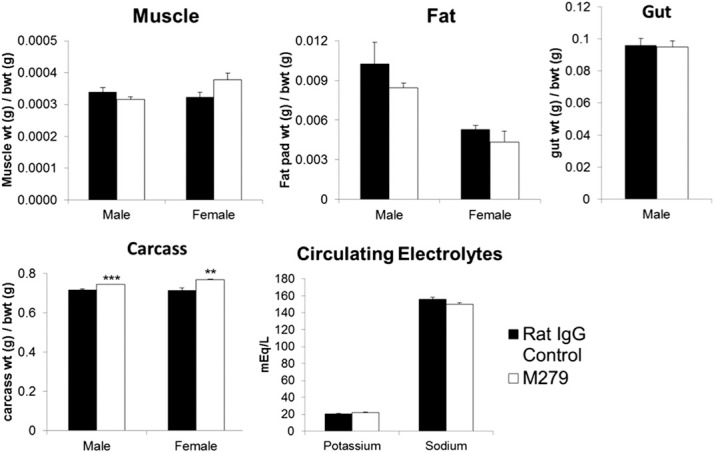
Effect of anti-CSF1R antibody on tissues and circulating electrolytes. Mice were injected with 200 μg rat IgG control or M279, 3× weekly for 6 weeks. Graphs show the mean ± sem of the tissues indicated, the total carcass weight after removal of the viscera, or the serum electrolytes. Significance is indicated by **P < 0.01, and ***P < 0.001, using an unpaired *t*-test; *n* = 6–8 mice/group.

At the end of the treatments, the organs and tissues of each mouse were weighed individually. Hematological analysis revealed a small (5–10%) but significant (*P*<0.05) increase in RBC count and significant (*P*<0.01) decrease in mean red cell volume from 48.8 fl to 45.5 fl. WBC counts were not altered significantly (Supplemental Fig. 2). After 6 weeks of anti-CSF1R antibody treatment, the absolute weight of the liver was reduced, mirroring the increase in liver weight seen in CSF1-treated mice and supporting the proposed role of CSF1-dependent macrophages in liver homeostasis (unpublished results). Despite the overall increase in body weight, in the male mice, there was decrease in absolute spleen, liver, and kidney weight and organ weight:body weight ratio. The female mice also displayed significant reduction in their liver weight:body weight ratio (Fig. 1B). The increased body weight was not associated with any change in the weight of the soleus muscle or individual fat pads or the entire gastrointestinal tract. Indeed, there was only a marginal increase in total carcass weight in both sexes (**Fig. 2**). Hence, we conclude that the difference between the treated and control animals is a result of an increase in blood volume and/or body fluids. There was no obvious tissue edema nor any change in serum electrolytes (Fig. 2).

### Loss of OCL and increased bone volume in anti-CSF1R antibody-treated mice

**Figure 3.**
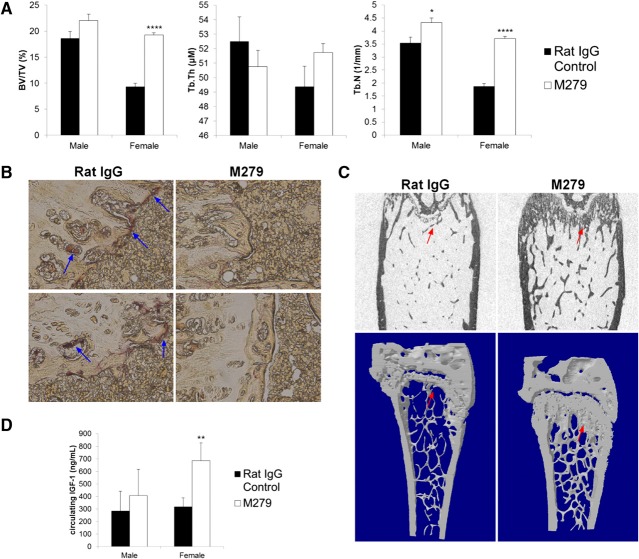
Effect of anti-CSF1R antibody on bone. Mice were injected with 200 μg rat IgG control or M279, 3× weekly for 6 weeks. The right femur from each mouse was harvested, analyzed for MicroCT scan, or prepared for histological examination, as described in Materials and Methods. (A) Graphs show the mean ± sem of the BV/TV, Tb.Th, or Tb.N, determined from the MicroCT scan. (B) TRAP+ cells (blue arrows) in growth plates. (C) Two-dimensional image (upper) and three-dimensional image (lower) of MicroCT scan from a treated (M279) and control (rat IgG) female, highlighting expansion of the growth plate (red arrows). (D) Circulating IGF1 ELISA. Significance is indicated by *P < 0.05, **P < 0.01, and ****P < 0.0001, using an unpaired *t*-test.

Osteopetrosis in *op/op* and *Csf1r*-deficient mice is attributed to an almost complete deficiency in bone-resorbing OCL [6]. CSF1 treatment of adult mice produced a substantial increase in OCL numbers (unpublished results) and transgenic overexpression of CSF1 produces osteoporosis [19], indicating that OCL production/difference remains sensitive to availability of CSF1. Bone density in mice declines with age but more rapidly in females [20], providing a natural and less-extreme model of osteoporosis compared with the commonly applied ovariectomy models. By the end of our experiments, the mice were 14–16 weeks of age. To determine whether the prolonged anti-CSF1R antibody treatment had an effect on bone, femurs from mice were collected, fixed, and analyzed by MicroCT. In keeping with the published data, **Fig. 3A** shows that the BV/TV and Tb.N were reduced by almost 50% in control female compared with male mice. The Tb.Th of remaining trabeculae was also reduced. In both male and female mice, TRAP-positive cells (OCL) were almost completely ablated around the growth plates of anti-CSF1R antibody-treated mice (Fig. 3B). In male mice, there was only a marginal increase in bone density and trabecular volume after 6 weeks of treatment, which was not significant. By contrast, the femurs from female anti-CSF1R-treated mice had clear increases in bone volume, as well as Tb.N. The impacts were visibly obvious in sections and MicroCT images, which also showed an expansion of the growth plate (Fig. 3C). In essence, the treated female mice were indistinguishable from the males, suggesting that anti-CSF1R completely prevents the age-dependent decline in bone density in female mice. Bone growth and density are directly regulated by circulating levels of IGF1 [21]. To investigate whether the dramatic increase in bone volume and Tb.N of anti-CSF1R antibody-treated females could be a result of increased IGF1, serum samples were analyzed. Antibody-treated females demonstrated a twofold increase in circulating IGF1 in their serum, whereas there was no significant change in the male mice (Fig. 3D). In summary, these mouse data suggest that anti-CSF1R treatment could have potential in preventing or reversing age-dependent osteoporosis.

### Effects of anti-CSF1R in the bone marrow

**Figure 4.**
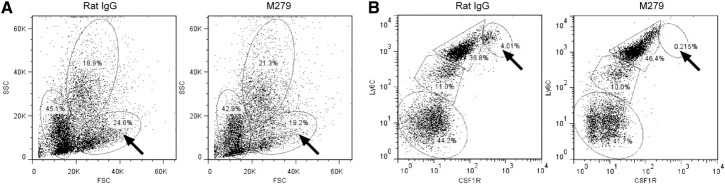
The effect of prolonged anti-CSF1R treatment on bone marrow cells. Representative FACS profiles of bone marrow cells derived from control rat IgG or anti-CSF1R treated mice. (A) FSC/SSC profiles highlighting the selective loss of the population of large cells with relatively low SSC (arrows). (B) CSF1R/Ly-6C profiles highlighting an almost complete loss of cells with high-surface CSF1R and Ly6C (arrows).

As noted previously, anti-CSF1R treatment did not alter the abundance of blood monocytes, suggesting that CSF1R signaling is not absolutely required for monocytopoiesis. The treatment does impair development of the Ly6C^lo^ monocyte subset [10–12]. We wished to determine whether these effects on monocyte differentiation actually occurred in the marrow and also whether there was any impact on progenitors that could also contribute to the loss of OCL. A recent report identified OCL progenitors among the Ly6C^hi^ monocyte-like population in marrow [22]. The anti-CSF1R mAb-treated mice produced a small but reproducible effect on the FSC and SSC profiles of bone marrow (**Fig. 4A**), selectively reducing the large (FSC^hi^), less granular (SSC^lo^) population by ∼5%. However, the antibody clearly ablated the Ly-6C^hi^/CSF1R^hi^ population, from 4.8% of total cells in control to only 0.5% in treated mice (*P*<0.0001), which was partly balanced by an apparent increase in the Ly6C^hi^/CSF1R^int^ in anti-CSF1R mAb-treated mice (Fig. 4B). In summary, even prolonged treatment with anti-CSF1R did not appear to interfere greatly with monocyte production or maturation but may have removed candidate OCL progenitors.

### Pleiotropic effects of prolonged anti-CSF1R treatment

If anti-CSF1R is to be used therapeutically in chronic disease situations, then it is important to consider the possible side-effects of macrophage depletion. The *op/op* mouse has a number of severe development defects, and the deletion of the *Csf1r* produces perinatal lethality. We therefore wished to determine whether any of these effects might be paralleled by a continuing requirement for CSF1R signaling in the adult. One of these effects of CSF1 deficiency is the loss of pancreatic β cells and insulin production [23]. However, the prolonged anti-CSF1R treatment, which ablates macrophage populations in the pancreas and adipose tissue all over the body [10], had no effect on average size or β-cell distribution within islets of Langerhans detected by immunostaining for insulin (Supplemental Fig. 3).

**Figure 5.**
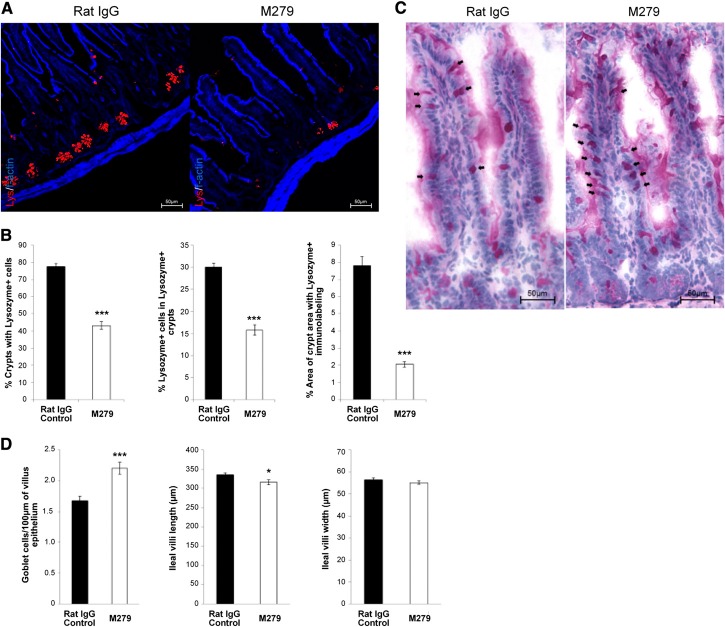
Effect of anti-CSF1R treatment on Paneth and goblet cells of the intestine. (A) Four-percent, PFA-fixed, 5 μm cryosections of ileum from control (rat IgG) or anti-CSF1R-treated (M279) mice were immunostained to detect lysozyme (red) in granules of Paneth cells within intestinal crypts, counterstained with F-actin (blue); original scale bars, 50 μm. (B) Morphometric analysis was performed to identify the proportion of crypts with Lysozyme^+^ staining, proportion of Lysozyme^+^ cells within Lysozyme^+^ crypts, and overall immunolabeling of lysozyme within crypt regions. Graphs show the mean ± sem for the comparative difference between control (rat IgG) and anti-CSF1R (M279) treatment. Significance is indicated by *P < 0.05, and ***P < 0.001, as determined by Mann-Whitney U-tests. Twenty villi/mouse were counted (*n*=8 mice). (C) Four-percent, PFA-fixed, 5 μm paraffin-embedded sections were analyzed for positive PAS staining for goblet cells in control (rat IgG) and anti-CSF1R-treated (M279) mice. (D) Ileal intestine sections. Morphometric analysis was performed to analyze PAS^+^ cells/100 μm villus; average ileal villus length and average villus width.

The *op/op* mouse and *Csf1r*-deficient mice also have substantial defects in intestinal proliferation and differentiation, including a loss of leucine-rich repeat-containing G protein-coupled receptor 5-positive epithelial progenitors and of Paneth cells [24–26]. Although these effects have been attributed to direct roles of CSF1 on epithelial cells, the *Csf1r-*EGFP transgene is not detectable in the crypts. *Csf1r*-EGFP-positive macrophages are abundant in the lamina propria and in intimate contact with these structures (Supplemental Fig. 1). Following prolonged treatment with anti-CSF1R, the Paneth cell density within crypts of Lieberkühn, highlighted by staining of lysozyme, was reduced substantially (**Fig. 5A** and **B**). Conversely, and in keeping with the phenotype of the *op/op* mouse [24–26], there a small increase in goblet cell density, identified by PAS staining (PAS^+^ cells; Fig. 5C). Despite these changes and the complete loss of the abundant *Csf1r*-EGFP-positive macrophage population from the lamina propria (Supplemental Fig. 1), there was no evidence of deficient epithelial renewal. Average villus length was marginally reduced in anti-CSF1R-treated mice, whereas villus width (combined mean of three cross-sections of each villus from top, bottom, and middle) was unchanged (Fig. 5D).

**Figure 6.**
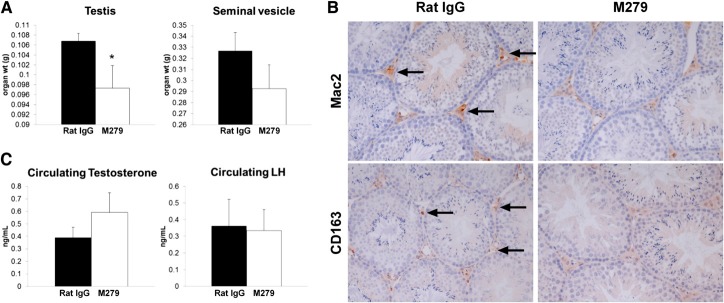
Effect of anti-CSF1R treatment on the testis. Mice were injected with 200 μg rat IgG control or M279, 3× weekly for 6 weeks. Serum was collected, as well as tissue, which was fixed and processed as described in Materials and Methods. Graphs show the mean ± sem. Significance is indicated by *P < 0.05, using unpaired *t*-tests. (A) Testis and seminal vesicle weight. (B) Representative sections show macrophages within the testicular interstitium, stained with a macrophage marker, Mac-2 or CD163. (C) Serum levels of testosterone and LH.

The *op/op* mouse is both male- and female-infertile [3, 4]. In female mice, the antibody did not deplete *Csf1r*-EGFP-positive macrophages from the ovary or uterus. We also examined the estrous cycle stage of the female mice when they were killed and noted a range of cycle stages. It is possible that the treatment would alter the duration of the cycle, but this has not been tested systematically. We focused instead on the male mice. The *op/op* mouse has deficient testosterone production, lower levels of LH, and a failure of the normal testosterone-feedback pathway in the hypothalamus [27, 28]. Other approaches to macrophage depletion support a role for macrophages in Leydig cell function [29]. Three-week treatment with anti-CSF1R completely ablated the interstitial macrophages, as detected with the *Csf1r-*EGFP transgene [10]. There was significant reduction in testis weight following the prolonged 6 weeks of anti-CSF1R antibody treatment (**Fig. 6A**). The fixation required for ultrastructural preservation precludes the use of the *Csf1r*-EGFP transgene directly for localization, so immunohistochemistry was used. The complete removal of macrophages in the testicular interstitium by prolonged anti-CSF1R treatment was confirmed using Mac-2 or CD163 anti-macrophage antibodies (Fig. 6B). Nevertheless, the testicular architecture and spermatogenesis appeared completely unaffected by ablation of the macrophages. To assess specifically the impact on Leydig cell function, we assayed circulating hormones. There was no difference in either circulating testosterone or LH concentrations (Fig. 6C), and seminal vesicle weight, a biomarker of androgen signaling, was also unchanged between control and treated animals (Fig. 6A).

### Lack of anti-CSF1R antibody toxicity and pathology

Despite the drastic decrease in resident tissue macrophages in many of the organs of the anti-CSF1R antibody-treated mice, H&E sections of skin, small intestine, pancreas, lung, liver, kidney, and spleen revealed no overt pathology (data not shown). In light of the loss of macrophages in the gut and liver, we considered the possibility that there would be exposure of the liver cells to contents from the gut or other impacts on liver cell function. Aside from the regulation of hepatocyte proliferation described previously, Kupffer cells have been ascribed many roles in clearance and protection of the body from potentially toxic metabolites in the portal blood [30]. Gene-expression arrays were used to determine whether there were any changes in hepatic gene expression associated with the evident decrease in macrophages. In the entire dataset comparing replicated treated and untreated samples, there were no transcripts that were differentially increased by >1.5-fold at an adjusted *P* value of 0.05. Kupffer cells are a relatively small proportion of total mRNA in the liver, and macrophage-specific transcripts are on the boundaries of detection. We examined the array data for evidence of depletion of macrophages. Transcripts, such as *Cd163*, *Csf1r*, *Siglec1* (which encodes sialic acid-binding Ig-like lectin 1), and those encoding class II MHC (*H2-Ab1*) and *Emr1* [which encodes epidermal growth factor-like module containing mucin-like receptor 1 (F4/80)], showed a decline of ∼50% (*P*<0.05; Supplemental Fig. 4). The residual activity is likely a result of monocytes and granulocytes in the blood, as these livers were not cleared before removal into Trizol.

## DISCUSSION

This study extends our earlier report on the impact of treatment of mice with a blocking anti-CSF1R antibody [10]. The prolonged treatment uncovered physiological impacts of macrophage depletion of clinical relevance, notably the accelerated weight gain after 3 weeks (Fig. 1) and the prevention of bone loss (Fig. 3). But even after the 6 weeks of macrophage depletion, some functions that are deficient in the *op/op* mouse were unaffected. As discussed in detail elsewhere [3], the M279 antibody used here has subtly different effects from the more widely used AFS98 rat-anti mouse CSF1R antibody. A previous, prolonged study using AFS98 was carried out in the context of diabetes in the *db/db* mouse, where 6 weeks of treatment was beneficial in preventing renal pathology, but the impact on the control mice was not tested [31]. Both antibodies block CSF1 and IL-34 binding with high affinity, but AFS98 causes much more rapid depletion of tissue macrophages, possibly via direct toxicity [3]. Many companies have produced small molecule inhibitors of CSF1R kinase activity; and most of these have been claimed to be highly specific [3]. However, given the high level of conservation of the tyrosine kinase domains of the type III protein tyrosine kinases (CSF1R, Fms-like tyrosine kinase-3, KIT, platelet-derived growth factor receptor), it would be difficult to predict off-target impacts in vivo, based on the in vitro data. The anti-CSF1R antibody effects can potentially provide the benchmark for effects that are likely to be genuinely on-target, as well as a potential therapy in its own right. The relatively slow depletion of macrophages seen with anti-CSF1R treatment suggests immediately that efficacy in a clinical scenario would require sustained treatment.

The definitive phenotype of the CSF1-deficient mouse is osteopetrosis, as a result of a complete lack of OCL [6, 32]. Additionally, toothless (*tl/tl*), a CSF1 null mutation in the rat, has few OCL and undetectable bone resorption, and the phenotype can be reversed with exogenous CSF1 [33]. In humans, variation at the *CSF1* locus has been implicated in bone loss in Paget's disease [34]. Many different CSF1R kinase inhibitors have been tested on various mouse disease models with some effect on bone: JNJ-28312141 prevented tumor-induced osteoclastogenesis and bone erosion [35]; Ki20227 inhibited osteolytic bone destruction through the suppression of CSF1-induced OCL accumulation in vivo [36]; SU11248 caused in vivo inhibition of osteolysis [37]; and one of the most-used CSF1R inhibitors, GW2580, completely inhibited bone degradation in cultures of human OCLs, rat calvaria, and rat fetal long bone [38]. More recently, yet another inhibitor, PLX3397, attenuated osteoclastic bone resorption in a model of neurofibromatosis [39]. None of these studies has applied treatment to natural age-associated bone loss in mice, and all have potential off-target effects via other kinases. Figure 3 shows that anti-CSF1R treatment completely ablated the TRAP-positive OCL populations in mouse bone and greatly reduced their Ly6C^hi^, CSF1R^hi^ candidate progenitors (Fig. 4). The loss of OCL did not greatly increase bone density in male mice, suggesting that the impact is balanced by a decrease in bone formation. Paradoxically, like anti-CSF1R, CSF1 treatment of mice can also increase bone density, despite increased OCL number (unpublished results) perhaps because of the interaction between bone-lining macrophages and osteoblasts [40, 41]. However, anti-CSF1R treatment completely prevented the substantial decrease in bone density seen in the female mice. The effect could be partially a result of the increased, circulating IGF1 (Fig. 3), although the levels of IGF1 in controls were the same in males and females and therefore, not correlated to bone density. Local and systemic human rIGF1 treatment increased new bone formation [42]. Anti-CSF1R treatment has been considered as a means of removing macrophages from tumors [10]; an impact of treatment upon hypercalcemia of malignancy [43] could be a secondary benefit. It will be of some interest to determine whether, like antibodies against the receptor activator of NF-κB ligand, anti-CSF1R can block glucocorticoid-induced bone loss [44].

Aside from the impacts on bone, the prolonged anti-CSF1R treatment was surprisingly well-tolerated. Where anti-CSF1 treatment has been shown to impair postnatal somatic growth [17] and CSF1 treatment to promote it [45], the treatment of the sexually mature, young-adult mice with anti-CSF1R did not compromise their continued weight gain. So, the CSF1-dependent phase of somatic growth occurs before the liver becomes the major source of IGF1 at sexual maturity. Anti-CSF1R did reduce the size of the liver, thereby supporting our hypothesis that CSF1 contributes to the homeostatic regulation of liver size (unpublished results) and consistent with the report that the *op/op* mouse cannot regenerate the liver following partial hepatectomy [46]. We do not have an explanation for the apparent fluid accumulation in the treated mice, which did not appear to be associated with any overt pathology.

Several other reported phenotypes of the CSF1 and CSF1R-deficient mice probably reflect nonredundant roles of macrophages in development rather than homeostasis. For example, we saw no effect of anti-CSF1R on β-cell number in the pancreatic islets (Supplemental Fig. 3). The close physical and functional relationship between testicular macrophages and Leydig cells in control of testosterone production is well-described. One of the major roles of macrophages under normal circumstances is to generate and provide 25-hydroxy-cholesterol as a substrate for steroid hormone production [29]. The impact of the *op/op* mouse implicates CSF1 as a key factor in male and female reproductive development [27–29, 47]. Our data now suggest that macrophage support of steady-state testis steroidogenesis in adulthood is largely dispensable or at least can be compensated through the LH/testosterone feedback.

The *op/op* mouse also has deficiencies in intestinal differentiation [24–26]. These reports identified numerous alterations in villus architecture, resembling the morphology of the villi reported in patients with malabsorption and lipid-engorgement disorders. The appearance of the mutant villi was also abnormal, and the average number of cells/crypt and cells/villus was reduced significantly in both mutants. Such pathologies would be of significant concern in the application of anti-CSF1R treatment. However, whereas the prolonged treatment greatly reduced the numbers of Paneth cells and produced a small increase in goblet cell number, there was no sign of altered villus architecture. The intestinal phenotype in the *op/op* mouse has been attributed to expression of CSF1R within the villus. In one study, *Csf1r*^fl/fl^ were crossed to mice expressing a tamoxifen-inducible *Cre* transgene driven by an intestine-specific promoter (VillinCre^*ERT2*^). Tamoxifen treatment produced slow ablation of the Paneth cells. These studies did not include a control with an irrelevant Cre target gene other than *Csf1r*, so direct effects of overexpression of Cre recombinase on stem-cell proliferation and/or survival cannot be eliminated. The *Csf1r-*EGFP reporter gene is not expressed at all in the crypt, but macrophages are in intimate contact with the basement membrane (Supplemental Fig. 1). Furthermore, analysis of the large gene-expression datasets produced by the FANTOM5 consortium [48] indicates that there is no detectable expression of *Csf1r* mRNA within isolated crypts. Although CSF1 has been reported to promote clonogenic growth of murine colonic crypt preparations [49], the more recent studies on the colon also argue that CSF1 acts indirectly to promote colonic enterocyte proliferation [25]. Accordingly, we suggest that the effect of the anti-CSF1R antibody on Paneth cells is an indirect consequence of the depletion of the lamina propria macrophages.

Others have claimed that the CSF1R is expressed functionally in subsets of neurons [50] and in renal proximal tubule cells [51]. In both cases, CSF1 has been attributed to roles in regeneration and/or cytoprotection. As in the intestine, these claims are not supported by the pattern of expression of the *Csf1r*-EGFP transgene [5] nor is *Csf1r* mRNA expression detected in isolated neurons, crypts, or any purified epithelial cells based on deep 5′RACE-tag sequencing of mouse or human [48]. We saw no effect of systemic anti-CSF1R treatment on microglial cell numbers in the brain nor any apparent effect on renal architecture, despite the complete loss of the large interstitial macrophage population.

In conclusion, our studies suggest that many of the effects of *Csf1* and *Csf1r* mutations reflect development roles that are redundant or functionally compensated in an adult mouse. The effect on bone turnover suggests that the treatment could have potential in the treatment of osteoporosis and may be well-tolerated. Of course, the important caveat to this study is that macrophages are an important component of the innate immune system and immune homeostasis. There is the potential to make inflammatory processes considerably worse with anti-CSF1R treatment [10]. So, there is a need to explore further the impacts of this treatment on host defense.

## Supplementary Material

Supplemental Data

## References

[1] Chitu V. Stanley E. R. (2006) Colony-stimulating factor-1 in immunity and inflammation. Curr. Opin. Immunol. 18, 39–48. 16337366 10.1016/j.coi.2005.11.006

[2] Hume D. A. (2006) The mononuclear phagocyte system. Curr. Opin. Immunol. 18, 49–53. 16338128 10.1016/j.coi.2005.11.008

[3] Hume D. A. MacDonald K. P. (2012) Therapeutic applications of macrophage colony-stimulating factor-1 (CSF-1) and antagonists of CSF-1 receptor (CSF-1R) signaling. Blood 119, 1810–1820. 22186992 10.1182/blood-2011-09-379214

[4] Pollard J. W. (2009) Trophic macrophages in development and disease. Nat. Rev. Immunol. 9, 259–270. 19282852 10.1038/nri2528 PMC3648866

[5] Sasmono R. T. Oceandy D. Pollard J. W. Tong W. Pavli P. Wainwright B. J. Ostrowski M. C. Himes S. R. Hume D. A. (2003) A macrophage colony-stimulating factor receptor-green fluorescent protein transgene is expressed throughout the mononuclear phagocyte system of the mouse. Blood 101, 1155–1163. 12393599 10.1182/blood-2002-02-0569

[6] Dai X. M. Ryan G. R. Hapel A. J. Dominguez M. G. Russell R. G. Kapp S. Sylvestre V. Stanley E. R. (2002) Targeted disruption of the mouse colony-stimulating factor 1 receptor gene results in osteopetrosis, mononuclear phagocyte deficiency, increased primitive progenitor cell frequencies, and reproductive defects. Blood 99, 111–120. 11756160 10.1182/blood.v99.1.111

[7] Wang Y. Szretter K. J. Vermi W. Gilfillan S. Rossini C. Cella M. Barrow A. D. Diamond M. S. Colonna M. (2012) IL-34 is a tissue-restricted ligand of CSF1R required for the development of Langerhans cells and microglia. Nat. Immunol. 13, 753–760. 22729249 10.1038/ni.2360 PMC3941469

[8] Nandi S. Gokhan S. Dai X. M. Wei S. Enikolopov G. Lin H. Mehler M. F. Stanley E. R. (2012) The CSF-1 receptor ligands IL-34 and CSF-1 exhibit distinct developmental brain expression patterns and regulate neural progenitor cell maintenance and maturation. Dev. Biol. 367, 100–113. 22542597 10.1016/j.ydbio.2012.03.026 PMC3388946

[9] Bartocci A. Mastrogiannis D. S. Migliorati G. Stockert R. J. Wolkoff A. W. Stanley E. R. (1987) Macrophages specifically regulate the concentration of their own growth factor in the circulation. Proc. Natl. Acad. Sci. USA 84, 6179–6183. 2819867 10.1073/pnas.84.17.6179 PMC299033

[10] MacDonald K. P. Palmer J. S. Cronau S. Seppanen E. Olver S. Raffelt N. C. Kuns R. Pettit A. R. Clouston A. Wainwright B. (2010) An antibody against the colony-stimulating factor 1 receptor depletes the resident subset of monocytes and tissue- and tumor-associated macrophages but does not inhibit inflammation. Blood 116, 3955–3963. 20682855 10.1182/blood-2010-02-266296

[11] Yona S. Kim K. W. Wolf Y. Mildner A. Varol D. Breker M. Strauss-Ayali D. Viukov S. Guilliams M. Misharin A. (2013) Fate mapping reveals origins and dynamics of monocytes and tissue macrophages under homeostasis. Immunity 38, 79–91. 23273845 10.1016/j.immuni.2012.12.001 PMC3908543

[12] Hashimoto D. Chow A. Greter M. Saenger Y. Kwan W. H. Leboeuf M. Ginhoux F. Ochando J. C. Kunisaki Y. van Rooijen N. (2011) Pretransplant CSF-1 therapy expands recipient macrophages and ameliorates GVHD after allogeneic hematopoietic cell transplantation. J. Exp. Med. 208, 1069–1082. 21536742 10.1084/jem.20101709 PMC3092347

[13] Hashimoto D. Chow A. Noizat C. Teo P. Beasley M. B. Leboeuf M. Becker C. D. See P. Price J. Lucas D. (2013) Tissue-resident macrophages self-maintain locally throughout adult life with minimal contribution from circulating monocytes. Immunity 38, 792–804. 23601688 10.1016/j.immuni.2013.04.004 PMC3853406

[14] Ramaswamy S. Marshall G. R. McNeilly A. S. Plant T. M. (2000) Dynamics of the follicle-stimulating hormone (FSH)-inhibin B feedback loop and its role in regulating spermatogenesis in the adult male rhesus monkey (Macaca mulatta) as revealed by unilateral orchidectomy. Endocrinology 141, 18–27. 10614619 10.1210/endo.141.1.7276

[15] Corker C. S. Davidson D. W. (1978) A radioimmunoassay for testosterone in various biological fluids without chromatography. J. Steroid Biochem. 9, 373–374. 96306 10.1016/0022-4731(78)90634-9

[16] Dai X. M. Zong X. H. Sylvestre V. Stanley E. R. (2004) Incomplete restoration of colony-stimulating factor 1 (CSF-1) function in CSF-1-deficient Csf1op/Csf1op mice by transgenic expression of cell surface CSF-1. Blood 103, 1114–1123. 14525772 10.1182/blood-2003-08-2739

[17] Wei S. Lightwood D. Ladyman H. Cross S. Neale H. Griffiths M. Adams R. Marshall D. Lawson A. McKnight A. J. (2005) Modulation of CSF-1-regulated post-natal development with anti-CSF-1 antibody. Immunobiology 210, 109–119. 16164017 10.1016/j.imbio.2005.05.005

[18] Gow D. J. Sester D. P. Hume D. A. (2010) CSF-1, IGF-1, and the control of postnatal growth and development. J. Leukoc. Biol. 88, 475–481. 20519640 10.1189/jlb.0310158

[19] Wei S. Dai X. M. Stanley E. R. (2006) Transgenic expression of CSF-1 in CSF-1 receptor-expressing cells leads to macrophage activation, osteoporosis, and early death. J. Leukoc. Biol. 80, 1445–1453. 16973889 10.1189/jlb.0506304

[20] Glatt V. Canalis E. Stadmeyer L. Bouxsein M. L. (2007) Age-related changes in trabecular architecture differ in female and male C57BL/6J mice. J. Bone Miner. Res. 22, 1197–1207. 17488199 10.1359/jbmr.070507

[21] Yakar S. Rosen C. J. Beamer W. G. Ackert-Bicknell C. L. Wu Y. Liu J. L. Ooi G. T. Setser J. Frystyk J. Boisclair Y. R. (2002) Circulating levels of IGF-1 directly regulate bone growth and density. J. Clin. Invest. 110, 771–781. 12235108 10.1172/JCI15463 PMC151128

[22] Charles J. F. Hsu L. Y. Niemi E. C. Weiss A. Aliprantis A. O. Nakamura M. C. (2012) Inflammatory arthritis increases mouse osteoclast precursors with myeloid suppressor function. J. Clin. Invest. 122, 4592–4605. 23114597 10.1172/JCI60920 PMC3533532

[23] Banaei-Bouchareb L. Gouon-Evans V. Samara-Boustani D. Castellotti M. C. Czernichow P. Pollard J. W. Polak M. (2004) Insulin cell mass is altered in Csf1op/Csf1op macrophage-deficient mice. J. Leukoc. Biol. 76, 359–367. 15178709 10.1189/jlb.1103591

[24] Akcora D. Huynh D. Lightowler S. Germann M. Robine S. de May J. R. Pollard J. W. Stanley E. R. Malaterre J. Ramsay R. G. (2013) The CSF-1 receptor fashions the intestinal stem cell niche. Stem Cell Res. 10, 203–212. 23314290 10.1016/j.scr.2012.12.001 PMC4096353

[25] Huynh D. Akcora D. Malaterre J. Chan C. K. Dai X. M. Bertoncello I. Stanley E. R. Ramsay R. G. (2013) CSF-1 receptor-dependent colon development, homeostasis and inflammatory stress response. PloS One 8, e56951. 23451116 10.1371/journal.pone.0056951 PMC3579891

[26] Huynh D. Dai X. M. Nandi S. Lightowler S. Trivett M. Chan C. K. Bertoncello I. Ramsay R. G. Stanley E. R. (2009) Colony stimulating factor-1 dependence of paneth cell development in the mouse small intestine. Gastroenterology 137, 136–144. 19303020 10.1053/j.gastro.2009.03.004 PMC2706482

[27] Cohen P. E. Chisholm O. Arceci R. J. Stanley E. R. Pollard J. W. (1996) Absence of colony-stimulating factor-1 in osteopetrotic (csfmop/csfmop) mice results in male fertility defects. Biol. Reprod. 55, 310–317. 8828834 10.1095/biolreprod55.2.310

[28] Cohen P. E. Hardy M. P. Pollard J. W. (1997) Colony-stimulating factor-1 plays a major role in the development of reproductive function in male mice. Mol. Endocrinol. 11, 1636–1650. 9328346 10.1210/mend.11.11.0009

[29] Hutson J. C. (2006) Physiologic interactions between macrophages and Leydig cells. Exp. Biol. Med. (Maywood) 231, 1–7. 16380639 10.1177/153537020623100101

[30] Ramadori G. Moriconi F. Malik I. Dudas J. (2008) Physiology and pathophysiology of liver inflammation, damage and repair. J. Physiol. Pharmacol. 59 (Suppl. 1), 107–117. 18802219

[31] Lim A. K. Ma F. Y. Nikolic-Paterson D. J. Thomas M. C. Hurst L. A. Tesch G. H. (2009) Antibody blockade of c-fms suppresses the progression of inflammation and injury in early diabetic nephropathy in obese db/db mice. Diabetologia 52, 1669–1679. 19466391 10.1007/s00125-009-1399-3

[32] Ryan G. R. Dai X. M. Dominguez M. G. Tong W. Chuan F. Chisholm O. Russell R. G. Pollard J. W. Stanley E. R. (2001) Rescue of the colony-stimulating factor 1 (CSF-1)-nullizygous mouse (Csf1(op)/Csf1(op)) phenotype with a CSF-1 transgene and identification of sites of local CSF-1 synthesis. Blood 98, 74–84. 11418465 10.1182/blood.v98.1.74

[33] Joseph B. K. Marks S. C. Jr. Hume D. A. Waters M. J. Symons A. L. (1999) Insulin-like growth factor-I (IGF-I) and IGF-I receptor (IGF-IR) immunoreactivity in normal and osteopetrotic (toothless, tl/tl) rat tibia. Growth Factors 16, 279–291. 10427502 10.3109/08977199909069146

[34] Albagha O. M. Visconti M. R. Alonso N. Langston A. L. Cundy T. Dargie R. Dunlop M. G. Fraser W. D. Hooper M. J. Isaia G. (2010) Genome-wide association study identifies variants at CSF1, OPTN and TNFRSF11A as genetic risk factors for Paget's disease of bone. Nat. Genet. 42, 520–524. 20436471 10.1038/ng.562 PMC3217192

[35] Manthey C. L. Johnson D. L. Illig C. R. Tuman R. W. Zhou Z. Baker J. F. Chaikin M. A. Donatelli R. R. Franks C. F. Zeng L. (2009) JNJ-28312141, a novel orally active colony-stimulating factor-1 receptor/FMS-related receptor tyrosine kinase-3 receptor tyrosine kinase inhibitor with potential utility in solid tumors, bone metastases, and acute myeloid leukemia. Mol. Cancer Ther. 8, 3151–3161. 19887542 10.1158/1535-7163.MCT-09-0255

[36] Ohno H. Kubo K. Murooka H. Kobayashi Y. Nishitoba T. Shibuya M. Yoneda T. Isoe T. (2006) A c-fms tyrosine kinase inhibitor, Ki20227, suppresses osteoclast differentiation and osteolytic bone destruction in a bone metastasis model. Mol. Cancer Ther. 5, 2634–2643. 17121910 10.1158/1535-7163.MCT-05-0313

[37] Murray L. J. Abrams T. J. Long K. R. Ngai T. J. Olson L. M. Hong W. Keast P. K. Brassard J. A. O'Farrell A. M. Cherrington J. M. (2003) SU11248 inhibits tumor growth and CSF-1R-dependent osteolysis in an experimental breast cancer bone metastasis model. Clin. Exp. Metastasis 20, 757–766. 14713109 10.1023/b:clin.0000006873.65590.68

[38] Conway J. G. McDonald B. Parham J. Keith B. Rusnak D. W. Shaw E. Jansen M. Lin P. Payne A. Crosby R. M. (2005) Inhibition of colony-stimulating-factor-1 signaling in vivo with the orally bioavailable cFMS kinase inhibitor GW2580. Proc. Natl. Acad. Sci. USA 102, 16078–16083. 16249345 10.1073/pnas.0502000102 PMC1276040

[39] He Y. Rhodes S. D. Chen S. Wu X. Yuan J. Yang X. Jiang L. Li X. Takahashi N. Xu M. (2012) c-Fms signaling mediates neurofibromatosis type-1 osteoclast gain-in-functions. PloS One 7, e46900. 23144792 10.1371/journal.pone.0046900 PMC3492362

[40] Alexander K. A. Chang M. K. Maylin E. R. Kohler T. Muller R. Wu A. C. Van Rooijen N. Sweet M. J. Hume D. A. Raggatt L. J. (2011) Osteal macrophages promote in vivo intramembranous bone healing in a mouse tibial injury model. J. Bone Miner. Res. 26, 1517–1532. 21305607 10.1002/jbmr.354

[41] Chang M. K. Raggatt L. J. Alexander K. A. Kuliwaba J. S. Fazzalari N. L. Schroder K. Maylin E. R. Ripoll V. M. Hume D. A. Pettit A. R. (2008) Osteal tissue macrophages are intercalated throughout human and mouse bone lining tissues and regulate osteoblast function in vitro and in vivo. J. Immunol. 181, 1232–1244. 18606677 10.4049/jimmunol.181.2.1232

[42] Fowlkes J. L. Thrailkill K. M. Liu L. Wahl E. C. Bunn R. C. Cockrell G. E. Perrien D. S. Aronson J. Lumpkin C. K. Jr., (2006) Effects of systemic and local administration of recombinant human IGF-I (rhIGF-I) on de novo bone formation in an aged mouse model. J. Bone Miner. Res. 21, 1359–1366. 16939394 10.1359/JBMR.060618 PMC2424402

[43] Clines G. A. (2011) Mechanisms and treatment of hypercalcemia of malignancy. Curr. Opin. Endocrinol. Diabetes Obes. 18, 339–346. 21897221 10.1097/MED.0b013e32834b4401

[44] Hofbauer L. C. Zeitz U. Schoppet M. Skalicky M. Schuler C. Stolina M. Kostenuik P. J. Erben R. G. (2009) Prevention of glucocorticoid-induced bone loss in mice by inhibition of RANKL. Arthritis Rheum. 60, 1427–1437. 19404943 10.1002/art.24445

[45] Alikhan M. A. Jones C. V. Williams T. M. Beckhouse A. G. Fletcher A. L. Kett M. M. Sakkal S. Samuel C. S. Ramsay R. G. Deane J. A. (2011) Colony-stimulating factor-1 promotes kidney growth and repair via alteration of macrophage responses. Am J. Pathol. 179, 1243–1256. 21762674 10.1016/j.ajpath.2011.05.037 PMC3157188

[46] Amemiya H. Kono H. Fujii H. (2011) Liver regeneration is impaired in macrophage colony stimulating factor deficient mice after partial hepatectomy: the role of M-CSF-induced macrophages. J. Surg. Res. 165, 59–67. 20031174 10.1016/j.jss.2009.08.008

[47] Cohen P. E. Zhu L. Pollard J. W. (1997) Absence of colony stimulating factor-1 in osteopetrotic (csfmop/csfmop) mice disrupts estrous cycles and ovulation. Biol. Reprod. 56, 110–118. 9002639 10.1095/biolreprod56.1.110

[48] Forrest A. R. (2014) A human gene expression atlas based upon promoter activity. Nature. 10.1038/nature13182 [Epub ahead of print].

[49] Ramsay R. G. Micallef S. J. Williams B. Lightowler S. Vincan E. Heath J. K. Mantamadiotis T. Bertoncello I. (2004) Colony-stimulating factor-1 promotes clonogenic growth of normal murine colonic crypt epithelial cells in vitro. J. Interferon Cytokine Res. 24, 416–427. 15296653 10.1089/1079990041535638

[50] Luo J. Elwood F. Britschgi M. Villeda S. Zhang H. Ding Z. Zhu L. Alabsi H. Getachew R. Narasimhan R. (2013) Colony-stimulating factor 1 receptor (CSF1R) signaling in injured neurons facilitates protection and survival. J. Exp. Med. 210, 157–172. 23296467 10.1084/jem.20120412 PMC3549715

[51] Menke J. Iwata Y. Rabacal W. A. Basu R. Yeung Y. G. Humphreys B. D. Wada T. Schwarting A. Stanley E. R. Kelley V. R. (2009) CSF-1 signals directly to renal tubular epithelial cells to mediate repair in mice. J. Clin. Invest. 119, 2330–2342. 19587445 10.1172/JCI39087 PMC2719924

