# Supplementary material for: Pleiotropic effects of extended blockade of CSF1R signaling in adult mice
Source: J Leukoc Biol. 2014 Aug;96(2):265–74. doi: 10.1189/jlb.2A0114-006R (PMC4378363; doi:10.1189/jlb.2A0114-006R)
Supplement: Supplemental Data [file supp_96_2_265__index.html]

Pleiotropic effects of extended blockade of CSF1R signaling in adult mice — Supplemental Data 

# Pleiotropic effects of extended blockade of CSF1R signaling in adult mice

## Supplemental Data

**Files in this Data Supplement:**

- Supplemental Data - (*jlb.2A0114-006RSuppData.zip; compressed file 908 KB*)
