# Supplementary material for: Pleiotropic effects of extended blockade of CSF1R signaling in adult mice
Source: J Leukoc Biol. 2014 Aug;96(2):265–74. doi: 10.1189/jlb.2A0114-006R (PMC4378363; doi:10.1189/jlb.2A0114-006R)
Supplement: Supplemental Data [file supp_jlb.2A0114-006R_jlb.2A0114-006RSuppData.zip › jlb.2A0114-006RSuppFig1.pdf]

PLEIOTROPIC EFFECTS OF EXTENDED BLOCKADE OF CSF1R SIGNALLING IN ADULT MICE

Kristin A. Sauter[*], Clare Pridans[*], Anuj Sehgal[*], Yi Ting Tsai†, Barry M. Bradford[*], Sobia Raza[*], Lindsey Moffat[*], Deborah J. Gow[*], Philippa M. Beard[*], Neil A. Mabbott[*], Lee B. Smith† & David A. Hume[1]*

[*] The Roslin Institute and Royal (Dick) School of Veterinary Studies, University of Edinburgh, Easter Bush EH25 9RG, Scotland, UK.

† Medical Research Council Centre for Reproductive Health, University of Edinburgh, The Queen's Medical Research Institute, 47 Little France Crescent, Edinburgh, EH16 4TJ, Scotland, UK.

Summary

Prolonged anti-CSF1R prevents age-dependent bone loss in female mice but does not reproduce the phenotypic consequences of the CSF1R and CSF1 null mutations.

Key Words. Macrophage, osteoclast, bone, testis, Paneth, Kupffer, CSF1

Running Title. Effects of anti-CSF1R treatment in mice

Word Counts

Total character count	27, 508
Figures total	6 (+4 supplementary figures)
Colour Figures	3 (+2 supplementary figures)

References	51
Abstract	235 words
Summary sentence	23 words

[1] **Corresponding author**

Professor David A. Hume

The Roslin Institute

Easter Bush EH25 9RG

Phone +44-131-6519181

*Email: david.hume@roslin.ed.ac.uk

ABBREVIATIONS

BV/TV	bone volume over total volume (bone density)
CSF1	colony-stimulating factor 1
CSF1R	colony-stimulating factor 1 receptor
IGF1	insulin-like growth factor 1
Mab	monoclonal antibody
OCL	osteoclasts
PCV	packed cell volume
RBC	red blood cell
Tb.N	trabecular numbers
Tb.Th	trabecular thickness
TRAP	Tartrate-resistant acid phosphatase
R(D)SVS	Royal (Dick) School of Veterinary Studies
WBC	white blood cell

ABSTRACT

We investigated the role of colony-stimulating factor 1 receptor (CSF1R) signalling in adult mice using prolonged treatment with anti-CSF1R antibody. Mutation of the macrophage colony-stimulating factor (CSF1) gene in the *op/op* mouse produces numerous developmental abnormalities. Mutation of the CSF1 receptor has an even more penetrant phenotype, including perinatal lethality, because of the existence of a second ligand, IL34. These effects on development provide limited insight into functions of CSF1R signalling in adult homeostasis. The carcase weight and weight of several organs (spleen, kidney, and liver) were reduced in the treated mice, but overall body weight gain was increased. Despite the complete loss of Kupffer cells, there was no effect on liver gene expression. The treatment ablated osteoclasts, increased bone density and trabecular volume, and prevented the decline in bone mass seen in female mice with age. The *op/op* mouse has a deficiency in pancreatic beta cells, and in Paneth cells in gut wall. Only the latter was reproduced by the antibody treatment, and was associated with increased goblet cell number but no change in villus architecture. Male *op/op* mice are infertile due to testosterone insufficiency. Anti-CSF1R treatment ablated interstitial macrophages in the testis, but there was no sustained effect on testosterone or luteinising hormone. The results indicate an on-going requirement for CSF1R signalling in macrophage and osteoclast homeostasis, but indicate that most effects of CSF1 and CSF1R mutations are due to effects on development.

INTRODUCTION

Macrophage colony-stimulating factor (CSF1) controls the proliferation, differentiation, maturation and survival of cells of cells of the mononuclear phagocyte system [1-4]. The effects of CSF1 are mediated through the CSF1R, a protein tyrosine kinase receptor. Expression of *Csf1r* mRNA is myeloid-restricted in adult animals, and a *Csf1r*-EGFP reporter gene provides a convenient marker for macrophage-lineage cells in transgenic mice [5]. A natural mutation of the *Csf1* gene in mice (*op/op*) produces a reduction in macrophage numbers in most tissues of the body, accompanied by severe growth retardation, osteopetrosis, and deficiencies in sensory, reproductive, and other endocrine systems [3, 4]. A null mutation of *Csf1r* produces even more penetrant phenotypes, including significant postnatal mortality [6]. A second ligand for *Csf1r,* IL34, provides an explanation for the greater impact of receptor depletion. IL34 appears to be required for the generation of specific macrophage populations, notably microglia in the brain and epidermal Langerhans cells [7, 8].

CSF1 is not available in saturating concentrations in an adult mouse; the concentration is regulated in part by macrophage-mediated clearance in the liver and spleen providing a relatively simple homeostatic control of macrophage numbers [9]. Treatment of mice with CSF1 causes a massive increase in tissue macrophage numbers throughout the body [10]. Surprisingly, this leads to a rapid increase in the size of the liver and hepatocyte proliferation, suggesting a function for CSF1-dependent macrophages in liver homeostasis [10]. Conversely, continuous CSF1R signalling is required for the maintenance of macrophage populations in adult mice. The administration of a blocking monoclonal antibody (Mab) against the CSF1R gradually eliminated resident tissue macrophages from many different organs. The antibody did not prevent monocytopoieisis, but apparently prevented maturation of monocytes in peripheral blood to form the non-classical $Ly6C^-$

population [11, 12]. Consequently, the treatment did not prevent macrophage recruitment into inflammatory sites, indeed the treatment exacerbated pathology in a model of graft-versus-host disease. These basic findings were subsequently repeated using a different Mab [13]. The relatively slow depletion of tissue macrophages in the treated mice suggests that the major effect of anti-CSF1R is to prevent their replacement, either via recruitment or local self-renewal. This view has recently been confirmed in models in which tissue macrophages have been acutely-depleted and replenishment is blocked by anti-CSF1R [14]. The slow turnover of tissue macrophages means that after three weeks of treatment used previously, mice had only been fully-depleted for a short period and the consequences may not have become evident. Accordingly, in the current study, we aimed to define the impact of long-term treatment with anti-CSF1R.

MATERIALS AND METHODS

Ethics Statement

All animal work was reviewed and approved by ethical review panel at The Roslin Institute and The University of Edinburgh and conducted under the authority of Home Office Project Licences 60/3828 and 60/4259.

In vivo studies

M279 is a rat IgG2b Mab which blocks CSF1 and IL34 binding to the CSF1R. In previous studies, the optimal dose of M279 was determined by injecting with increasing doses thrice weekly for three weeks and serum samples taken and assayed by ELISA for circulating CSF1. A dose of 125-400 µg per injection had a maximal increase in circulating CSF1[11]. Accordingly, in all the studies detailed herein, mice were treated with 200 µg rat anti-mouse CSF1R antibody (M279, Amgen) or rat IgG (Sigma, I4131) administered by intraperitoneal injection, thrice weekly for 6 weeks. MacGreen EGFP$^+$ and EGFP$^-$ on the C57BL/6 background, as well as non-transgenic C57BL/6 male and female mice were treated starting at 8-9 weeks of age and weighed on each injection day. Following treatment, the C57BL/6 non-transgenic mice were sacrificed by CO_2 asphyxiation, and peripheral blood immediately collected by cardiac puncture into EDTA tubes (Teklab, K1230), organs and tissues were collected and weighed, animals were eviscerated and a carcass weight was measured. Both femurs were collected. Bone marrow was flushed from one femur for cellularity and cell subset analysis. The other femur was fixed overnight in formalin and stored in 70% ethanol.

Flow cytometry

Bone marrow was flushed from a femur from each animal, mechanically disrupted by pipetting, counted, and diluted to 1 X 10^6 cells in 100µl PBS. 200µl of 1 X 10^6 bone marrow

cells was stained in the dark at room temperature for 1 hour at 4°C with the following antibodies: APC anti-mouse CD115 (CSF1R) (clone AFS98, Biolegend), PerCP/Cy5.5 anti-mouse Ly-6C (clone HK1.4, Biolegend), Data were collected on a CyAn™ ADP Analyzer (Beckman Coulter), analysed using FlowJo 7.5.5 Flow Cytometry Analysis Software (TreeStar) and Minitab 16.1.0 (Minitab).

ELISA

Blood from EDTA tubes was centrifuged at 1000 x g at room temperature for 15 minutes. Plasma was collected and stored at -20°C. Insulin-like growth factor 1 (IGF1) ELISA was preformed according to manufacturer's instructions (Immunodiagnostic Systems, AC-18F1).

Immunohistochemistry

Organs were collected at cull, fixed in 4% paraformaldehyde and embedded in paraffin wax. Testis sections for immunohistochemistry were deparaffinised, rehydrated and antigen retrieved using a citrate buffer epitope retrieval method (10 Psi (0.68 atm), 125°C, 30 min, in citrate buffer pH= 6.0) before blocking of endogenous peroxidase and nonspecific binding sites. For single colour immuno-detection, primary antibodies (Anti-Mac 2, Cedarlane labs, Cat. No. CL8942AP; CD163 (M-96), Santa Cruz Biotechnology, inc., Cat. No. sc-33560; CD68, Abcam, Cat. No. ab955), were applied individually in normal horse serum, and incubated at 4°C for 24 hours, followed by incubation for secondary detection (Vector, immpressTM, Reagent Kit, Cat. No. MP-7401(Rabbit-CD163), 7402(Mouse-CD68), 7404(Rat-Mac2)), for one hour. Samples were washed in TBS, and DAB detection (Vector, ImmPACT™ Peroxidase Substrates, Cat. No. SK-4105) was used to resolve sites of immunolocalisation, while hematoxylin was used as counterstain. Sections were then mounted for downstream analysis. And visualised using

an Olympus Research Microscope AX70 Provis, Scotia, NY, USA, software: AxioVision Rel.4.8).

Bones were decalcified, embedded, and sectioned by the R(D)SVS Clinical Pathology Laboratory. Post-fixation and µCT scan, femurs and tibias were decalcified in 14% EDTA pH7.0 for 3 days at room temperature and embedded in paraffin wax. Serial sections 4µM thick were cut from each block and dried overnight at 37°C before a final drying at 60°C for 25 minutes. Sections were de-waxed in xylene, rehydrated through ethanol and washed. TRAP staining using Acid Phosphatase, leukocyte (TRAP) kit (387-A, Sigma) was carried out according to manufacturer's protocol, except incubation time was increased to 2 hours and a counterstain was not used.

For detection of lysozyme in intestinal crypts, 5µM cryosections from paraformaldehyde fixed tissues were permeabilized with 50% methanol for 20 minutes before immuonostaining with rabbit monoclonal anti-lysozyme antibody (Abcam, Cambridge) then Alexa-Flour 594-anti-rabbit IgG (Invitrogen, Paisley). Sections were counterstained with Alexa-647 Phalloidin (Dako, Ely) and examined using a Zeiss LSM5 confocal microscope (Zeiss, Welwyn Garden City). For quantification of goblet cells and analysis of general intestine villi morphology, sections were treated with periodic acid, then stained in Schiff's reagent (0.5% pararosaniline, 1% sodium metabisulfite; PAS staining) and counterstained with haematoxylin. For dual colour labelling of pancreas, anti-insulin antibody (Abcam, Cat. No. ab7842) was applied at 4°C for 24 hours before secondary detection (Biotinylated Goat Anti-Guinea Pig IgG Antibody, Vector, Cat. No. BA-7000). Immunofluorescence was visualised using the Perkin Elmer, TSA™ Plus Cyanine 3 System, Cat. No. NEL744B001KT, following manufacturer's instructions). Anti-PDX1 antibody (Abcam, Cat. No. ab47267) was also applied in normal horse serum, and secondary detection system (Vector, immpressTM, Reagent Kit, Cat. No. MP-7401(Rabbit)) was used. Finally,

Cytogreen was use as counterstain and the sections were mounted for confocal analysis (Zeiss, LSM710, software : Zen2011).

Analysis of bone architecture by microcomputed tomography (MicroCT)

MicroCT analysis was performed at the left distal femoral using a Skyscan 1172 instrument set (Kontlich, Belgium) at 60 kV, and 150 µA at a resolution of 5µm. The images were reconstructed using the Skyscan NRecon program and analysed using Skyscan CTAn software. A volume of 200 slices was measured 100µm proximal to the primary spongiosa.

Complete blood count analysis

Blood was collected into 0.5ml EDTA tubes (Teklab). Total WBC, RBC, PCV were all measured on the ABX Pentra 60 haematology analyser. WBC differential counts were performed by making a blood smear and counterstaining with Giemsa stain prior to counting.

Electrolytes

Serum electrolytes (Na and K) were measured using an Instrumentation laboratory IL650 analyser using ion selective electrodes.

Testosterone and luteinising hormone (LH) Hormone assays

Quantification of circulating hormones was carried out as previously described [15, 16].

RNA extraction, labelling, and microarray hybridisation

Total RNA was extracted using TRIzol® reagent (Invitrogen) from liver tissue homogenised in Lysing Matrix D using a FastPrep Instrument (Q biogene). RNA was eluted in water and its quality was assessed with an Agilent 2100 Bioanalyser (Agilent

Technologies) before being used for labelling. Microarray was performed by Edinburgh Genomics at The Roslin Institute. Total RNA (50ng) was amplified using the Nugen Pico SL kit, 2.5µg of the resulting cDNA was biotin labelled using the Nugen Encore labelling kit using the half volume protocol. The biotin labelled product was prepared for hybridisation according to the Nugen protocol for Affymetrix Gene Titan hybridisation, using the Affymetrix Gene Titan Hybridisation Wash & Stain Kit for WT Array Plates (PN 901622). The samples were hybridized to Affymetrix Mouse Gene 1.1 ST Array plates using the appropriate Hyb-Wash-Scan protocol for this plate and the Gene Titan Hyb Wash Stain kit for the reagents (Affymetrix). Image generation and the resulting CEL files for analysis were produced in Affymetrix® GeneChip® Command Console® Software (AGCC) version 3.0.1. Initial QCs were performed in Expression Console. The obtained AffymetrixCEL files were imported into the Genomics Suite software package version 6.13.0213 (Partek Inc). GEO: GSE53573.

Statistical analysis

Data on all graphs were analysed using an unpaired t-test or Mann-Whitney nonparametric test as indicated. Results are presented as the mean and standard error of the mean (SEM). All analyses were performed using GraphPad Prism 5.0 (GraphPad Software Inc, San Diego, CA). A P value < 0.05 was considered significant.

RESULTS

The effects of prolonged treatment with Anti-CSF1R Mab M279.

Age and weight-matched groups of male and female mice were treated with M279, a blocking anti-CSF1R antibody [11], or a rat IgG control three times weekly for six weeks. CSF1-deficient mice have severe postnatal growth retardation [3] which can be restored by administration of CSF1 [17] and mimicked by anti-CSF1 treatment in the postnatal period [18]. We have shown elsewhere that CSF1-dependent macrophages are significant source of IGF1 in the postnatal period [19]. After the onset of puberty, the major source of IGF1 for growth is the liver. The adult mice of both sexes continued to gain weight regardless of anti-CSF1R treatment. Indeed, by the beginning of week three, weight gain in the treated mice showed a clear acceleration relative to controls, and the faster rate of relative weight gain continued throughout the final 4 weeks in the antibody-treated animals (Fig. 1A).

The efficacy of the anti-CSF1R treatment was confirmed as a nearly complete reduction of *Csf1r*-EGFP-positive macrophages in the spleen and intestine (Supplementary Fig. 1) extending the findings from the shorter 3 week treatment [11]. The extended treatment did not produce any more extensive depletion in sites such as lung and uterus, in which macrophage populations appeared independent of CSF1R signalling [11]. Some additional organs have been analysed in detail below. In one of the experimental series we examined an additional group of mice 3 weeks after cessation of their 6 week anti-CSF1R treatment. Surprisingly, the *Csf1r*-EGFP-positive macrophage numbers in these mice showed no evidence of recovery, although the differential growth rate slowed (Supplementary Fig. 1). However, we do not have an independent measure of the clearance of the antibody in these mice so we do not know whether CSF1R signalling was restored.

At the end of the treatments, the organs and tissues of each mouse were individually weighed. Haematological analysis revealed a small (5-10%) but significant ($p<0.05$) increase in red blood cell count, and significant ($p<0.01$) decrease in mean red cell volume from 48.8fl to 45.5fl. WBC counts were not significantly altered (Supplementary Fig. 2). After six weeks of anti-CSF1R Ab treatment, the absolute weight of the liver was reduced, mirroring the increase in liver weight seen in CSF1-treated mice and supporting the proposed role of CSF1-dependent macrophages in liver homeostasis [10]. Despite the overall increase in body weight, in the male mice there was decrease in both absolute spleen, liver, and kidney weight and organ weight/body weight ratio. The female mice also displayed significant reduction in their liver weight/body weight ratio (Fig. 1B). The increased body weight was not associated with any change in the weight of the soleus muscle or individual fat pads or the entire gastrointestinal tract. Indeed, there was only a marginal increase in total carcass weight in both sexes (Fig. 2). Hence, we conclude that the difference between the treated and control animals is due to an increase in blood volume and/or body fluids. There was no obvious tissue oedema, nor any change in serum electrolytes (Fig. 2).

Loss of osteoclasts and increased bone volume in anti-CSF1R Ab-treated mice

Osteopetrosis in both *op/op* and *Csf1r*$^{-/-}$ mice is attributed to an almost complete deficiency in bone-resorbing osteoclasts (OCL) [6]. CSF1 treatment of adult mice produced a substantial increase in OCL numbers [10] and transgenic over-expression of CSF1 produces osteoporosis [20] indicating that OCL production/difference remains sensitive to availability of CSF1. Bone density in mice declines with age, but more rapidly in females [21] providing a natural, and less extreme model of osteoporosis compared to the commonly-applied ovariectomy models. By the end of our experiments, the mice were 14-16 weeks of age. To determine whether the prolonged anti-CSF1R antibody treatment had

an effect on bone, femurs from mice were collected, fixed, and analysed by µCT. In keeping with the published data, Fig. 3A shows that the bone volume (BV/TV) and trabecular numbers (Tb.N) were reduced by almost 50% in control female compared to male mice. The trabecular thickness (Tb.Th) of remaining trabeculae was also reduced. In both male and female mice, TRAP+ cells (OCLs) were almost completed ablated around the growth plates of anti-CSF1R Ab-treated mice (Fig. 3B). In male mice, there was only a marginal increase in bone density and trabecular volume after 6 weeks of treatment, which was not significant. By contrast, the femurs from female anti-CSF1R-treated mice had clear increases in bone volume as well as trabecular number (Tb.N). The impacts were visibly obvious in sections and µCT images, which also showed an expansion of the growth plate (Fig. 3C). In essence, the treated female mice were indistinguishable from the males, suggesting that anti-CSF1R completely prevents the age-dependent decline in bone density in female mice. Bone growth and density are directly regulated by circulating levels of IGF1 [22]. To investigate whether the dramatic increase in bone volume and trabecular number of anti-CSF1R Ab-treated females could be due to increased IGF1, serum samples were analysed. Antibody-treated females demonstrated a 2-fold increase in circulating IGF1 in their serum while there was no significant change in the male mice (Fig. 3D). In summary, these mouse data suggest that anti-CSF1R treatment could have potential in preventing or reversing age-dependent osteoporosis.

Effects of anti-CSF1R in the bone marrow

As noted previously, anti-CSF1R treatment did not alter the abundance of blood monocytes, suggesting that CSF1R signalling is not absolutely required for monocytopoiesis. The treatment does impair development of the Ly6C^{Lo} monocyte subset [11-13]. We wished to determine whether these effects on monocyte differentiation actually occurred in the marrow, and also whether there was any impact on progenitors that could

also contribute to the loss of OCL. A recent report identified OCL progenitors amongst the Ly6C^{Hi} monocyte-like population in marrow [23]. The anti-CSF1R MAb treated mice produced a small but reproducible effect on the forward (FSC) and side (SSC) scatter profiles of bone marrow (Fig. 4A), selectively reducing the large (FSC^{hi}), less granular (SSC^{lo}) population by around 5%. However, the antibody clearly ablated the Ly-6C^{Hi}/CSF1R^{Hi} population; from 4.8 % of total cells in control to only 0.5% in treated mice ($p<0.0001$), which was partly balanced by an apparent increase in the Ly6C^{hi}/CSF1R^{Int} in anti-CSF1R MAb treated mice (Fig. 4B). In summary, even prolonged treatment with anti-CSF1R did not appear to greatly interfere with monocyte production or maturation, but may have removed candidate OCL progenitors.

Pleiotropic effects of prolonged anti-CSF1R treatment.

If anti-CSF1R is to be used therapeutically in chronic disease situations, it is important to consider the possible side effects of macrophage depletion. The *op/op* mouse has a number of severe development defects, and the deletion of the *Csf1r* produces perinatal lethality. We therefore wished to determine whether any of these effects might be paralleled by a continuing requirement for CSF1R signalling in the adult. One of these effects of CSF1 deficiency is the loss of pancreatic beta cells and insulin production [24]. However, the prolonged anti-CSF1R treatment, which ablates macrophage populations in the pancreas and adipose tissue all over the body [11] had no effect on average size or beta-cell distribution within islets of Langerhans detected by immunostaining for insulin (Supplementary Fig. 3).

The *op/op* mouse and *Csf1r* $^{-/-}$ mice also have substantial defects in intestinal proliferation and differentiation including a loss of Lgr5-positive epithelial progenitors and of Paneth cells [25-27]. Although these effects have been attributed to direct roles of CSF1 on epithelial cells, the *Csf1r*-EGFP transgene is not detectable in the crypts. *Csf1r*-EGFP-

positive macrophages are abundant in the lamina propria and in intimate contact with these structures (Supplementary Fig. 1). Following prolonged treatment with anti-CSF1R, the Paneth cell density within crypts of Lieberkühn, highlighted by staining of lysozyme, was substantially reduced (Fig. 5A,B). Conversely, and in keeping with the phenotype of the *op/op* mouse [25-27], there a small increase in goblet cell density, identified by PAS staining (PAS^+ cells) (Fig. 5C). Despite these changes, and the complete loss of the abundant *Csf1r*-EGFP-positive macrophage population from the lamina propria (Supplementary Fig. 1) there was no evidence of deficient epithelial renewal. Average villus length was marginally reduced in anti-CSF1R treated mice whilst villus width (combined mean of 3 cross sections of each villus from top, bottom and middle) was unchanged (Fig. 5D).

The *op/op* mouse is both male and female infertile [3, 4]. In female mice, the antibody did not deplete *Csf1r*-EGFP-positive macrophages from the ovary or uterus. We also examined the estrous cycle stage of the female mice when they were sacrificed, and noted a range of cycle stages. It is possible that the treatment would alter the duration of the cycle, but this has not been tested systematically. We focussed instead on the male mice. The *op/op* mouse has deficient testosterone production, lower levels of luteinising hormone (LH), and a failure of the normal testosterone feedback pathway in the hypothalamus [28, 29]. Other approaches to macrophage depletion support a role for macrophages in Leydig cell function [30]. Three week treatment with anti-CSF1R completely ablated the interstitial macrophages as detected with the *Csf1r*-EGFP transgene [11]. There was significant reduction in testis weight following the prolonged 6 week anti-CSF1R Ab treatment (Fig. 6A). The fixation required for ultrastructural preservation precludes the use of the *Csf1r*-EGFP transgene directly for localisation, so immunohistochemistry was employed. The complete removal of macrophages in the testicular interstitium by prolonged anti-CSF1R treatment was confirmed using Mac2 or

ED2 anti-macrophage antibodies (Fig. 6B). Nevertheless, the testicular architecture and spermatogenesis appeared completely unaffected by ablation of the macrophages. To specifically assess the impact on Leydig cell function we assayed circulating hormones. There was no difference in either circulating testosterone or LH concentrations (Fig. 6C), and seminal vesicle weight, a biomarker of androgen signalling was also unchanged between control and treated animals (Fig. 6A).

Lack of anti-CSF1R Ab toxicity and pathology

Despite the drastic decrease in resident tissue macrophages in many of the organs of the anti-CSF1R Ab-treated mice, H&E sections of skin, small intestine, pancreas, lung, liver, kidney, and spleen revealed no overt pathology (data not shown). In the light of the loss of macrophages in the gut and liver, we considered the possibility that there would be exposure of the liver cells to contents from the gut, or other impacts on liver cell function. Aside from the regulation of hepatocyte proliferation described previously, Kupffer cells have been ascribed many roles in clearance and protection of the body from potentially toxic metabolites in the portal blood [31]. Gene expression arrays were used to determine whether there were any changes in hepatic gene expression associated with the evident decrease in macrophages. In the entire dataset comparing replicated treated and untreated samples, there were no transcripts that were differentially increased by >1.5 fold at an adjusted p value of 0.05. Kupffer cells are a relatively small proportion of total mRNA in the liver and macrophage-specific transcripts are on the boundaries of detection. We examined the array data for evidence of depletion of macrophages. Transcripts such as *Cd163, Csf1r, Siglec1,* those encoding class II MHC *(H2-Ab1)* and *Emr1* (F4/80) showed a decline of around 50% ($p<0.05$) (Supplementary Fig. 4). The residual activity is likely due to monocytes and granulocytes in the blood; since these livers were not cleared prior to removal into Trizol®.

DISCUSSION

This study extends our earlier report on the impact of treatment of mice with a blocking anti-CSF1R antibody [11]. The prolonged treatment uncovered physiological impacts of macrophage depletion of clinical relevance, notably the accelerated weight gain after 3 weeks (Fig. 1) and the prevention of bone loss (Fig. 3). But even after the six weeks of macrophage depletion, some functions that are deficient in the op/op mouse were unaffected. As discussed in detail elsewhere [3], the M279 antibody used here has subtly different effects from the more widely-used AFS98 rat-anti mouse CSF1R antibody. A previous prolonged study using AFS98 was carried out in the context of diabetes in the *db/db* mouse, where 6 weeks of treatment was beneficial in preventing renal pathology, but the impact on the control mice was not tested [32]. Both antibodies block CSF1 and IL34 binding with high affinity, but AFS98 causes much more rapid depletion of tissue macrophages, possibly via direct toxicity [3]. Many companies have produced small molecule inhibitors of CSF1R kinase activity; and most of these have been claimed to highly-specific [3]. However, given the high level of conservation of the tyrosine kinase domains of the type III protein tyrosine kinases (CSF1R, FLT3, KIT, PDGFR) it would be difficult to predict off target impacts *in vivo* based upon the *in vitro* data. The anti-CSF1R antibody effects can potentially provide the benchmark for effects that are likely to be genuinely on-target, as well as a potential therapy in its own right. The relatively slow depletion of macrophages seen with anti-CSF1R treatment immediately suggests that efficacy in a clinical scenario would require sustained treatment.

The definitive phenotype of the CSF1-deficient mouse is osteopetrosis due to a complete lack of OCL [6, 33]. Additionally, toothless (*tl/tl*), a CSF1 null mutation in the rat, has few OCL and undetectable bone resorption, and the phenotype can be reversed with exogenous CSF1 [34]. In humans, variation at the CSF1 locus has been implicated in

bone loss in Paget's disease [35]. Many different CSF1R kinase inhibitors have been tested on various mouse disease models with some effect on bone. JNJ-28312141 prevented tumor-induced OCLogenesis and bone erosion [36]. Ki20227 inhibited osteolytic bone destruction through the suppression of CSF1-induced OCL accumulation *in vivo* [37]. SU11248 caused *in vivo* inhibition of osteolysis [38] and one of the most-used CSF1R inhibitors, GW2580 completely inhibited bone degradation in cultures of human OCLs, rat calvaria, and rat fetal long bone [39]. More recently yet another inhibitor, PLX3397, attenuated OCLic bone resorption in a model of neurofibromatosis [40]. None of these studies has applied treatment to natural age-associated bone loss in mice, and all have potentially off target effects via other kinases. Fig. 3 shows that anti-CSF1R treatment completely ablated the TRAP-positive OCL populations in mouse bone, and greatly reduced their Ly6C^{Hi}, CSF1R^{Hi} candidate progenitors (Fig. 4). The loss of OCL did not greatly increase bone density in male mice, suggesting that the impact is balanced by a decrease in bone formation. Paradoxically, like anti-CSF1R, CSF1 treatment of mice can also increase bone density despite increased OCL number [10], perhaps because of the interaction between bone-lining macrophages and osteoblasts [41, 42]. However, anti-CSF1R treatment completely prevented the substantial decrease in bone density seen in the female mice. The effect could be partially due to the increased circulating IGF1 (Fig. 3), although the levels of IGF1 in controls were the same in males and females and therefore not correlated to bone density. Local and systemic rhIGFI treatment increased new bone formation [43]. Anti-CSF1R treatment has been considered as a means of removing macrophages from tumours [11]; an impact of treatment upon hypercalcemia of malignancy [44] could be a secondary benefit. It will be of some interest to determine whether, like antibodies against RANKL, anti-CSF1R can block glucocorticoid-induced bone loss [45].

Aside from the impacts on bone, the prolonged anti-CSF1R treatment was surprisingly well-tolerated. Where anti-CSF1 treatment has been shown to impair postnatal somatic growth [18] and CSF1 treatment to promote it [46], the treatment of the sexually mature young adult mice with anti-CSF1R did not compromise their continued weight gain. So, the CSF1-dependent phase of somatic growth occurs before the liver becomes the major source of IGF1 at sexual maturity. Anti-CSF1R did reduce the size of the liver, thereby supporting our hypothesis that CSF1 contributes to the homeostatic regulation of liver size [10] and consistent with the report that the *op/op* mouse cannot regenerate the liver following partial hepatectomy [47]. We do not have an explanation for the apparent fluid accumulation in the treated mice, which did not appear to be associated with any overt pathology.

Several other reported phenotypes of the CSF1 and CSF1R-deficient mice probably reflect non-redundant roles of macrophages in development rather than homeostasis. For example, we saw no effect of anti-CSF1R on beta cell number in the pancreatic islets (Supplementary Fig. 3). The close physical and functional relationship between testicular macrophages and Leydig cells in control of testosterone production is well described. One of the major roles of macrophages under normal circumstances is to generate and provide 25-hydroxy-cholesterol as a substrate for steroid hormone production [30]. The impact of the *op/op* mouse implicates CSF1 as a key factor in male and female reproductive development [28-30, 48]. Our data now suggest that macrophage support of steady-state testis steroidogenesis in adulthood is largely dispensable, or at least can be compensated through the LH/testosterone feedback.

The *op/op* mouse also has deficiencies in intestinal differentiation [25-27]. These reports identified numerous alterations in villus architecture resembling the morphology of the villi reported in patients with malabsorption and lipid-engorgement disorders. The appearance

of the mutant villi was also abnormal, and the average number of cells per crypt and per villus was significantly reduced in both mutants. Such pathologies would be significant concerns in the application of anti-CSF1R treatment. However, whilst the prolonged treatment greatly reduced the numbers of Paneth cells and produced a small increase in Goblet cell number there was no sign of altered villus architecture. The intestinal phenotype in the *op/op* mouse has been attributed to expression of CSF1R within the villus. In one study, Csf1r$^{fl/fl}$ were crossed to mice expressing a tamoxifen-inducible *Cre*-transgene driven by an intestine-specific promoter (*VillinCre^{ERT2}*). Tamoxifen treatment produced slow ablation of the Paneth cells. These studies did not include a control with an irrelevant cre target gene other than *Csf1r*, so direct effects of over-expression of Cre recombinase on stem cell proliferation and/or survival cannot be eliminated. The *Csf1r*-EGFP reporter gene is not expressed at all in the crypt, but macrophages are in intimate contact with the basement membrane (Supplementary Fig. 1). Furthermore, analysis of the large gene expression datasets produced by the FANTOM5 Consortium [49] indicates that there is no detectable expression of *Csf1r* mRNA within isolated crypts. Although CSF1 has been reported to promote clonogenic growth of murine colonic crypt preparations [50], the more recent studies on colon also argue that CSF1 acts indirectly to promote colonic enterocyte proliferation [26]. Accordingly, we suggest that the effect of the anti-CSF1R antibody on Paneth cells is an indirect consequence of the depletion of the lamina propria macrophages.

Others have claimed that the CSF1 receptor is expressed functionally in subsets of neurons [51] and in renal proximal tubule cells [52]. In both cases, CSF1 has been attributed roles in regeneration and/or cytoprotection. As in the intestine, these claims are not supported by the pattern of expression of the *Csf1r*-EGFP transgene [5] nor is *Csf1r* mRNA expression detected in isolated neurons, crypts or any purified epithelial cells based upon deep 5'RACE tag sequencing of mouse or human [49]. We saw no effect of

systemic anti-CSF1R treatment on microglial cell numbers in the brain nor any apparent effect on renal architecture despite the complete loss of the large interstitial macrophage population.

In conclusion, our studies suggest that many of the effects of *Csf1* and *Csf1r* mutations reflect development roles that are either redundant, or functionally compensated, in an adult mouse. The effect on bone turnover suggests that the treatment could have potential in the treatment of osteoporosis and may be well-tolerated. Of course, the important caveat to this study is that macrophages are an important component of the innate immune system and immune homeostasis. There is the potential to make inflammatory processes considerably worse with anti-CSF1R treatment [11]. So, there is a need to explore further the impacts of this treatment on host defense.

AUTHORSHIP

DAH, KAS and CP conceived the study. KAS, CP, DJG, AS, YTT, BMB, SR, LM, PMB, NA. M, LBS & DAH designed the study, performed experiments, analysed data, and contributed to writing.

ACKNOWLEDGMENTS

This study was supported by project and Institute Strategic grant funding from the Biotechnology and Biological Sciences Research Council and MRC. We thank Bob Fleming, and staff in the Biological Research Facility, the Pathology Services Group and the R(D)SVS Clinical Pathology Laboratory (University of Edinburgh, UK) for excellent technical support. We thank Forbes Howie for technical support with hormone assays.

CONFLICT OF INTEREST DISCLOSURE

The authors declare no conflict of interest.

REFERENCES

1. Chitu V, Stanley ER: **Colony-stimulating factor-1 in immunity and inflammation**. *Current opinion in immunology* 2006, **18**(1):39-48.
2. Hume DA: **The mononuclear phagocyte system**. *Current Opinion in Immunology* 2006, **18**(1):49-53.
3. Hume DA, MacDonald KP: **Therapeutic applications of macrophage colony-stimulating factor-1 (CSF-1) and antagonists of CSF-1 receptor (CSF-1R) signaling**. *Blood* 2012, **119**(8):1810-1820.
4. Pollard JW: **Trophic macrophages in development and disease**. *Nature reviews Immunology* 2009, **9**(4):259-270.
5. Sasmono RT, Oceandy D, Pollard JW, Tong W, Pavli P, Wainwright BJ, Ostrowski MC, Himes SR, Hume DA: **A macrophage colony-stimulating factor receptor-green fluorescent protein transgene is expressed throughout the mononuclear phagocyte system of the mouse**. *Blood* 2003, **101**(3):1155-1163.
6. Dai XM, Ryan GR, Hapel AJ, Dominguez MG, Russell RG, Kapp S, Sylvestre V, Stanley ER: **Targeted disruption of the mouse colony-stimulating factor 1 receptor gene results in osteopetrosis, mononuclear phagocyte deficiency, increased primitive progenitor cell frequencies, and reproductive defects**. *Blood* 2002, **99**(1):111-120.
7. Wang Y, Szretter KJ, Vermi W, Gilfillan S, Rossini C, Cella M, Barrow AD, Diamond MS, Colonna M: **IL-34 is a tissue-restricted ligand of CSF1R required for the development of Langerhans cells and microglia**. *Nature immunology* 2012, **13**(8):753-760.
8. Nandi S, Gokhan S, Dai XM, Wei S, Enikolopov G, Lin H, Mehler MF, Stanley ER: **The CSF-1 receptor ligands IL-34 and CSF-1 exhibit distinct developmental brain expression patterns and regulate neural progenitor cell maintenance and maturation**. *Developmental biology* 2012, **367**(2):100-113.
9. Bartocci A, Mastrogiannis DS, Migliorati G, Stockert RJ, Wolkoff AW, Stanley ER: **Macrophages specifically regulate the concentration of their own growth factor in the circulation**. *Proceedings of the National Academy of Sciences of the United States of America* 1987, **84**(17):6179-6183.
10. Gow DJ, Sauter KA, Pridans C, Moffat L, Stutchfield BM, Raza S, Beard PM, Sehgal A, Tsai Y-T, Bainbridge G *et al*: **A novel CSF1-Fc conjugate expands mononuclear phagocyte populations and indirectly promotes extensive hepatocyte proliferation.** . *AmJPathol* 2014, **In press**.
11. MacDonald KP, Palmer JS, Cronau S, Seppanen E, Olver S, Raffelt NC, Kuns R, Pettit AR, Clouston A, Wainwright B *et al*: **An antibody against the colony-stimulating factor 1 receptor depletes the resident subset of monocytes and tissue- and tumor-associated macrophages but does not inhibit inflammation**. *Blood* 2010, **116**(19):3955-3963.

12. Yona S, Kim KW, Wolf Y, Mildner A, Varol D, Breker M, Strauss-Ayali D, Viukov S, Guilliams M, Misharin A *et al*: **Fate mapping reveals origins and dynamics of monocytes and tissue macrophages under homeostasis**. *Immunity* 2013, **38**(1):79-91.
13. Hashimoto D, Chow A, Greter M, Saenger Y, Kwan WH, Leboeuf M, Ginhoux F, Ochando JC, Kunisaki Y, van Rooijen N *et al*: **Pretransplant CSF-1 therapy expands recipient macrophages and ameliorates GVHD after allogeneic hematopoietic cell transplantation**. *The Journal of experimental medicine* 2011, **208**(5):1069-1082.
14. Hashimoto D, Chow A, Noizat C, Teo P, Beasley MB, Leboeuf M, Becker CD, See P, Price J, Lucas D *et al*: **Tissue-resident macrophages self-maintain locally throughout adult life with minimal contribution from circulating monocytes**. *Immunity* 2013, **38**(4):792-804.
15. Ramaswamy S, Marshall GR, McNeilly AS, Plant TM: **Dynamics of the follicle-stimulating hormone (FSH)-inhibin B feedback loop and its role in regulating spermatogenesis in the adult male rhesus monkey (Macaca mulatta) as revealed by unilateral orchidectomy**. *Endocrinology* 2000, **141**(1):18-27.
16. Corker CS, Davidson DW: **A radioimmunoassay for testosterone in various biological fluids without chromatography**. *Journal of steroid biochemistry* 1978, **9**(4):373-374.
17. Dai XM, Zong XH, Sylvestre V, Stanley ER: **Incomplete restoration of colony-stimulating factor 1 (CSF-1) function in CSF-1-deficient Csf1op/Csf1op mice by transgenic expression of cell surface CSF-1**. *Blood* 2004, **103**(3):1114-1123.
18. Wei S, Lightwood D, Ladyman H, Cross S, Neale H, Griffiths M, Adams R, Marshall D, Lawson A, McKnight AJ *et al*: **Modulation of CSF-1-regulated post-natal development with anti-CSF-1 antibody**. *Immunobiology* 2005, **210**(2-4):109-119.
19. Gow DJ, Sester DP, Hume DA: **CSF-1, IGF-1, and the control of postnatal growth and development**. *Journal of leukocyte biology* 2010, **88**(3):475-481.
20. Wei S, Dai XM, Stanley ER: **Transgenic expression of CSF-1 in CSF-1 receptor-expressing cells leads to macrophage activation, osteoporosis, and early death**. *Journal of leukocyte biology* 2006, **80**(6):1445-1453.
21. Glatt V, Canalis E, Stadmeyer L, Bouxsein ML: **Age-related changes in trabecular architecture differ in female and male C57BL/6J mice**. *Journal of bone and mineral research : the official journal of the American Society for Bone and Mineral Research* 2007, **22**(8):1197-1207.
22. Yakar S, Rosen CJ, Beamer WG, Ackert-Bicknell CL, Wu Y, Liu JL, Ooi GT, Setser J, Frystyk J, Boisclair YR *et al*: **Circulating levels of IGF-1 directly regulate bone growth and density**. *The Journal of clinical investigation* 2002, **110**(6):771-781.
23. Charles JF, Hsu LY, Niemi EC, Weiss A, Aliprantis AO, Nakamura MC: **Inflammatory arthritis increases mouse osteoclast precursors with myeloid suppressor function**. *The Journal of clinical investigation* 2012, **122**(12):4592-4605.
24. Banaei-Bouchareb L, Gouon-Evans V, Samara-Boustani D, Castellotti MC, Czernichow P, Pollard JW, Polak M: **Insulin cell mass is altered in Csf1op/Csf1op macrophage-deficient mice**. *Journal of leukocyte biology* 2004, **76**(2):359-367.
25. Akcora D, Huynh D, Lightowler S, Germann M, Robine S, de May JR, Pollard JW, Stanley ER, Malaterre J, Ramsay RG: **The CSF-1 receptor fashions the intestinal stem cell niche**. *Stem cell research* 2013, **10**(2):203-212.
26. Huynh D, Akcora D, Malaterre J, Chan CK, Dai XM, Bertoncello I, Stanley ER, Ramsay RG: **CSF-1 receptor-dependent colon development, homeostasis and inflammatory stress response**. *PloS one* 2013, **8**(2):e56951.
27. Huynh D, Dai XM, Nandi S, Lightowler S, Trivett M, Chan CK, Bertoncello I, Ramsay RG, Stanley ER: **Colony stimulating factor-1 dependence of paneth cell development in the mouse small intestine**. *Gastroenterology* 2009, **137**(1):136-144, 144 e131-133.
28. Cohen PE, Chisholm O, Arceci RJ, Stanley ER, Pollard JW: **Absence of colony-stimulating factor-1 in osteopetrotic (csfmop/csfmop) mice results in male fertility defects**. *Biology of reproduction* 1996, **55**(2):310-317.
29. Cohen PE, Hardy MP, Pollard JW: **Colony-stimulating factor-1 plays a major role in the development of reproductive function in male mice**. *Mol Endocrinol* 1997, **11**(11):1636-1650.

30. Hutson JC: **Physiologic interactions between macrophages and Leydig cells**. *Exp Biol Med (Maywood)* 2006, **231**(1):1-7.
31. Ramadori G, Moriconi F, Malik I, Dudas J: **Physiology and pathophysiology of liver inflammation, damage and repair**. *Journal of physiology and pharmacology : an official journal of the Polish Physiological Society* 2008, **59 Suppl 1**:107-117.
32. Lim AK, Ma FY, Nikolic-Paterson DJ, Thomas MC, Hurst LA, Tesch GH: **Antibody blockade of c-fms suppresses the progression of inflammation and injury in early diabetic nephropathy in obese db/db mice**. *Diabetologia* 2009, **52**(8):1669-1679.
33. Ryan GR, Dai XM, Dominguez MG, Tong W, Chuan F, Chisholm O, Russell RG, Pollard JW, Stanley ER: **Rescue of the colony-stimulating factor 1 (CSF-1)-nullizygous mouse (Csf1(op)/Csf1(op)) phenotype with a CSF-1 transgene and identification of sites of local CSF-1 synthesis**. *Blood* 2001, **98**(1):74-84.
34. Joseph BK, Marks SC, Jr., Hume DA, Waters MJ, Symons AL: **Insulin-like growth factor-I (IGF-I) and IGF-I receptor (IGF-IR) immunoreactivity in normal and osteopetrotic (toothless, tl/tl) rat tibia**. *Growth Factors* 1999, **16**(4):279-291.
35. Albagha OM, Visconti MR, Alonso N, Langston AL, Cundy T, Dargie R, Dunlop MG, Fraser WD, Hooper MJ, Isaia G *et al*: **Genome-wide association study identifies variants at CSF1, OPTN and TNFRSF11A as genetic risk factors for Paget's disease of bone**. *Nature genetics* 2010, **42**(6):520-524.
36. Manthey CL, Johnson DL, Illig CR, Tuman RW, Zhou Z, Baker JF, Chaikin MA, Donatelli RR, Franks CF, Zeng L *et al*: **JNJ-28312141, a novel orally active colony-stimulating factor-1 receptor/FMS-related receptor tyrosine kinase-3 receptor tyrosine kinase inhibitor with potential utility in solid tumors, bone metastases, and acute myeloid leukemia**. *Mol Cancer Ther* 2009, **8**(11):3151-3161.
37. Ohno H, Kubo K, Murooka H, Kobayashi Y, Nishitoba T, Shibuya M, Yoneda T, Isoe T: **A c-fms tyrosine kinase inhibitor, Ki20227, suppresses osteoclast differentiation and osteolytic bone destruction in a bone metastasis model**. *Molecular cancer therapeutics* 2006, **5**(11):2634-2643.
38. Murray LJ, Abrams TJ, Long KR, Ngai TJ, Olson LM, Hong W, Keast PK, Brassard JA, O'Farrell AM, Cherrington JM *et al*: **SU11248 inhibits tumor growth and CSF-1R-dependent osteolysis in an experimental breast cancer bone metastasis model**. *Clinical & experimental metastasis* 2003, **20**(8):757-766.
39. Conway JG, McDonald B, Parham J, Keith B, Rusnak DW, Shaw E, Jansen M, Lin P, Payne A, Crosby RM *et al*: **Inhibition of colony-stimulating-factor-1 signaling in vivo with the orally bioavailable cFMS kinase inhibitor GW2580**. *Proceedings of the National Academy of Sciences of the United States of America* 2005, **102**(44):16078-16083.
40. He Y, Rhodes SD, Chen S, Wu X, Yuan J, Yang X, Jiang L, Li X, Takahashi N, Xu M *et al*: **c-Fms signaling mediates neurofibromatosis Type-1 osteoclast gain-in-functions**. *PloS one* 2012, **7**(11):e46900.
41. Alexander KA, Chang MK, Maylin ER, Kohler T, Muller R, Wu AC, Van Rooijen N, Sweet MJ, Hume DA, Raggatt LJ *et al*: **Osteal macrophages promote in vivo intramembranous bone healing in a mouse tibial injury model**. *Journal of bone and mineral research : the official journal of the American Society for Bone and Mineral Research* 2011, **26**(7):1517-1532.
42. Chang MK, Raggatt LJ, Alexander KA, Kuliwaba JS, Fazzalari NL, Schroder K, Maylin ER, Ripoll VM, Hume DA, Pettit AR: **Osteal tissue macrophages are intercalated throughout human and mouse bone lining tissues and regulate osteoblast function in vitro and in vivo**. *J Immunol* 2008, **181**(2):1232-1244.
43. Fowlkes JL, Thrailkill KM, Liu L, Wahl EC, Bunn RC, Cockrell GE, Perrien DS, Aronson J, Lumpkin CK, Jr.: **Effects of systemic and local administration of recombinant human IGF-I (rhIGF-I) on de novo bone formation in an aged mouse model**. *Journal of bone and mineral research : the official journal of the American Society for Bone and Mineral Research* 2006, **21**(9):1359-1366.
44. Clines GA: **Mechanisms and treatment of hypercalcemia of malignancy**. *Current opinion in endocrinology, diabetes, and obesity* 2011, **18**(6):339-346.

45. Hofbauer LC, Zeitz U, Schoppet M, Skalicky M, Schuler C, Stolina M, Kostenuik PJ, Erben RG: **Prevention of glucocorticoid-induced bone loss in mice by inhibition of RANKL**. *Arthritis and rheumatism* 2009, **60**(5):1427-1437.
46. Alikhan MA, Jones CV, Williams TM, Beckhouse AG, Fletcher AL, Kett MM, Sakkal S, Samuel CS, Ramsay RG, Deane JA *et al*: **Colony-stimulating factor-1 promotes kidney growth and repair via alteration of macrophage responses**. *The American journal of pathology* 2011, **179**(3):1243-1256.
47. Amemiya H, Kono H, Fujii H: **Liver regeneration is impaired in macrophage colony stimulating factor deficient mice after partial hepatectomy: the role of M-CSF-induced macrophages**. *J Surg Res*, **165**(1):59-67.
48. Cohen PE, Zhu L, Pollard JW: **Absence of colony stimulating factor-1 in osteopetrotic (csfmop/csfmop) mice disrupts estrous cycles and ovulation**. *Biology of reproduction* 1997, **56**(1):110-118.
49. Forrest AR: **A human gene expression atlas based upon promoter activity**. *Nature in press* 2014.
50. Ramsay RG, Micallef SJ, Williams B, Lightowler S, Vincan E, Heath JK, Mantamadiotis T, Bertoncello I: **Colony-stimulating factor-1 promotes clonogenic growth of normal murine colonic crypt epithelial cells in vitro**. *Journal of interferon & cytokine research : the official journal of the International Society for Interferon and Cytokine Research* 2004, **24**(7):416-427.
51. Luo J, Elwood F, Britschgi M, Villeda S, Zhang H, Ding Z, Zhu L, Alabsi H, Getachew R, Narasimhan R *et al*: **Colony-stimulating factor 1 receptor (CSF1R) signaling in injured neurons facilitates protection and survival**. *The Journal of experimental medicine* 2013, **210**(1):157-172.
52. Menke J, Iwata Y, Rabacal WA, Basu R, Yeung YG, Humphreys BD, Wada T, Schwarting A, Stanley ER, Kelley VR: **CSF-1 signals directly to renal tubular epithelial cells to mediate repair in mice**. *The Journal of clinical investigation* 2009.

FIGURE LEGENDS

Fig. 1: Effect of anti-CSF1R antibody on body and organ weights

Mice were injected with 200 µg rat IgG control or M279 thrice weekly for 6 weeks. Graphs show the mean ± SEM. Significance is indicated by *$p<0.05$, **$p<0.01$, ***$p<0.001$, ****$p<0.0001$ using an unpaired t-test. n = 29 mice per group for body weight and n = 8 mice per group for organ weights. The groups were assigned to have similar average body weights at the outset. (A) average relative weight gain (normalised to Day 1) taken before each injection. (B) average spleen/body weight ratio, liver/body weight ratio, and kidney/body weight ratio at the end of treatment. Note that absolute body weight differences between the two groups are <4%, so the reduced organs weights also reflect absolute reductions.

Fig. 2: Effect of anti-CSF1R antibody on tissues and circulating electrolytes

Mice were injected with 200 µg rat IgG control or M279 thrice weekly for 6 weeks. Graphs show the mean ± SEM of the tissues indicated, the total carcass weight after removal of the viscera or the serum electrolytes. Significance is indicated by **p<0.01, ***p<0.001 using an unpaired t-test. n = 6-8 mice per group.

Fig. 3: Effect of anti-CSF1R antibody on bone.

Mice were injected with 200 µg rat IgG control or M279 thrice weekly for 6 weeks. The right femur from each mouse was harvested, analysed for µCT scan or prepared for histological examination as described in Materials and Methods. (A) Graphs show the mean ± SEM of the bone volume (BV/TV), trabecular thickness (Tb.Th) or trabecular number (Tb.N) determined from the µCT scan. (B) TRAP+ cells (blue arrows) in growth plates. (C) 2D image (upper) and 3D image (lower) of µCT scan from a treated (M279) and control (Rat IgG) female highlighting expansion of the growth plate (red arrows). (D) Circulating IGF-1 ELISA. Significance is indicated by *p<0.05, **p<0.01, ****p<0.0001 using an unpaired t-test.

Fig. 4: The effect of prolonged anti-CSF1R treatment on bone marrow cells

Representative FACS profiles of bone marrow cells derived from control rat IgG or anti-CSF1R treated mice. (A) FSC/SSC profiles highlighting the selective loss of the population of large cells with relatively low side scatter (arrows). (B) CSF-1R/Ly-6C profiles highlighting an almost complete loss of cells with high surface CSF1R and Ly6C (arrows).

Fig. 5: Effect of anti-CSF1R treatment on Paneth and Goblet cells of the intestine

(A) 4% PFA-fixed 5µm cryosections of ileum from control (Rat IgG) or anti-CSF1R-treated (M279) mice were immunostained to detect lysozyme (red) in granules of Paneth cells within intestinal crypts counterstained with F-actin (blue). Scale bar = 50µm. (B) Morphometric analysis was performed to identify the proportion of crypts with Lys^+

staining, proportion of Lys^+ positive cells within Lys^+ crypts and overall immunolabelling of lysozyme within crypts regions. Graphs show the mean ± SEM for the comparative difference between control (rat IgG) and anti-CSF1R (M279) treatment. Significance is indicated by *p<0.05 and ***p<0.001 as determined by Mann-Whitney U tests. 20 villi per mouse were counted (n=8 mice).(C) 4% PFA-fixed 5μm paraffin-embedded sections were analysed for positive PAS staining for goblet cells in control (rat IgG) and anti-CSF1R treated (M279) mice. (D) Ileal intestine sections. Morphometric analysis was performed to analyse PAS^+ cells per 100μm of villus, average ileal villus length and average villus width.

Fig. 6: Effect of anti-CSF1R treatment on the testis

Mice were injected with 200 μg rat IgG control or M279 thrice weekly for 6 weeks. Serum was collected as well as tissue, which was fixed and processed as described in Materials and Methods. Graphs show the mean ± SEM. Significance is indicated by *p<0.05 using unpaired t-tests. (A) Testis and seminal vesicle weight (B) Representative sections show macrophages within the testicular interstitium, stained with a macrophage marker, Mac2 or ED2. (C) Serum levels of testosterone and luteinising hormone (LH).

Fig. S1: Macrophage depletion with M279 treatment.

Mice were injected with 200 μg rat IgG control or M279 thrice weekly for 6 weeks. Cryosections were prepared from spleens (A) and the intestines (B). (C) Tiled confocal images of spleen cryosections from treated (M279) and control (Rat IgG) mice 3 weeks following the end of treatment. (D) Relative weight gain of treated (M279) and control (Rat IgG) at 6 and 9 weeks. Graph shows mean ± SEM. n=5.

Fig. S2: Blood analysis

Blood was analysed after 6 weeks of treatment and the percentage of white blood cells (left), total red blood cells (middle) and mean cell volume (right) determined. Graph shows mean ± SEM. n=5

Fig. S3: Immunofluorescence staining of pancreas

Cryosections of pancreas from control (Rat IgG) and treated (M279) mice were stained for insulin (red), PDX1 (blue) and counterstained with Cytogreen (green). Images are representative of 3 mice and 11 islets per group.

Fig. S4: Liver Microarray

Microarray analysis performed on treated (M279) and control (Rat IgG) liver. Graph shows mean expression ± SEM. n=3 (control) or 2 (M279). Significance is indicated by *$p<0.05$ using an unpaired t-test.

FIGURE 1

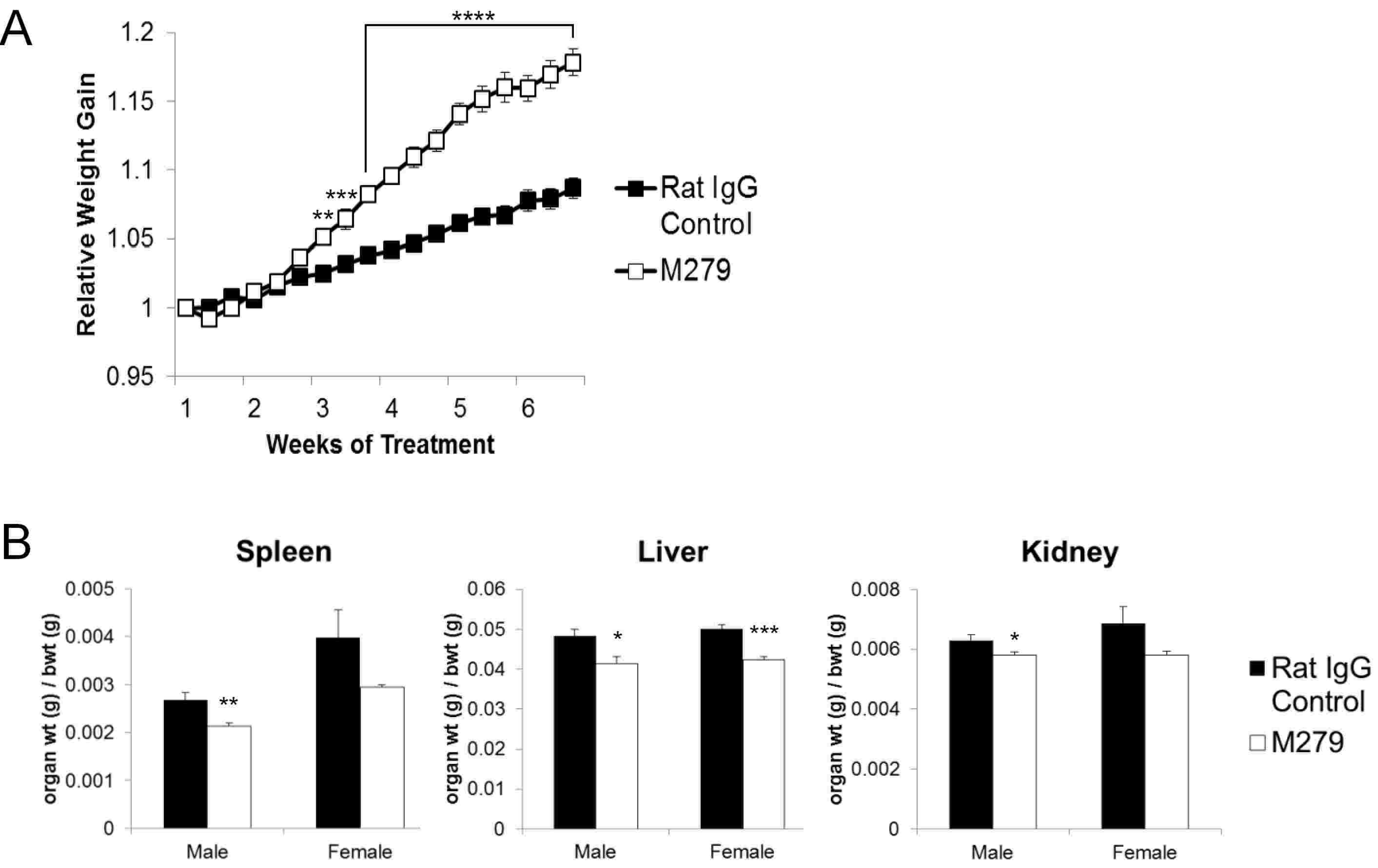
A
1.2
1.15
1.1
1.05
1
0.95
Relative Weight Gain
1
2
3
4
5
6
Weeks of Treatment
****
***
**
Rat IgG Control
M279
B
Spleen
Liver
Kidney
organ wt (g) / bwt (g)
0.005
0.004
0.003
0.002
0.001
0
0.06
0.05
0.04
0.03
0.02
0.01
0.008
0.006
0.004
0.002
Male
Female
*
Rat IgG Control
M279

FIGURE 2

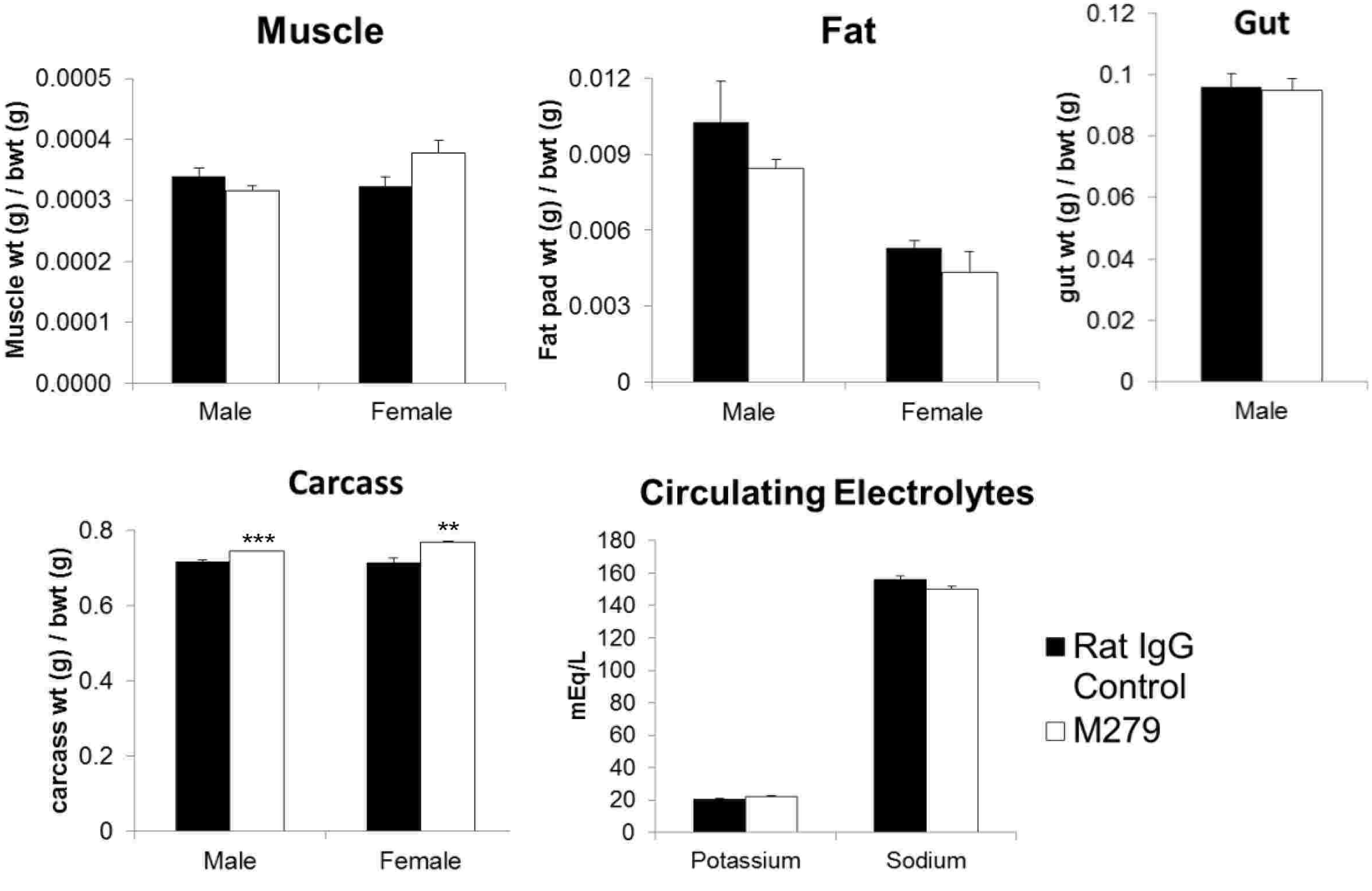

Muscle
Muscle wt (g) / bwt (g)
0.0005
0.0004
0.0003
0.0002
0.0001
0.0000
Male
Female
Fat
Fat pad wt (g) / bwt (g)
0.012
0.009
0.006
0.003
0
Male
Female
Gut
gut wt (g) / bwt (g)
0.12
0.1
0.08
0.06
0.04
0.02
0
Male
Carcass
carcass wt (g) / bwt (g)
0.8
0.6
0.4
0.2
0
***
**
Male
Female
Circulating Electrolytes
mEq/L
180
160
140
120
100
80
60
40
20
0
Potassium
Sodium
Rat IgG Control
M279

FIGURE 3

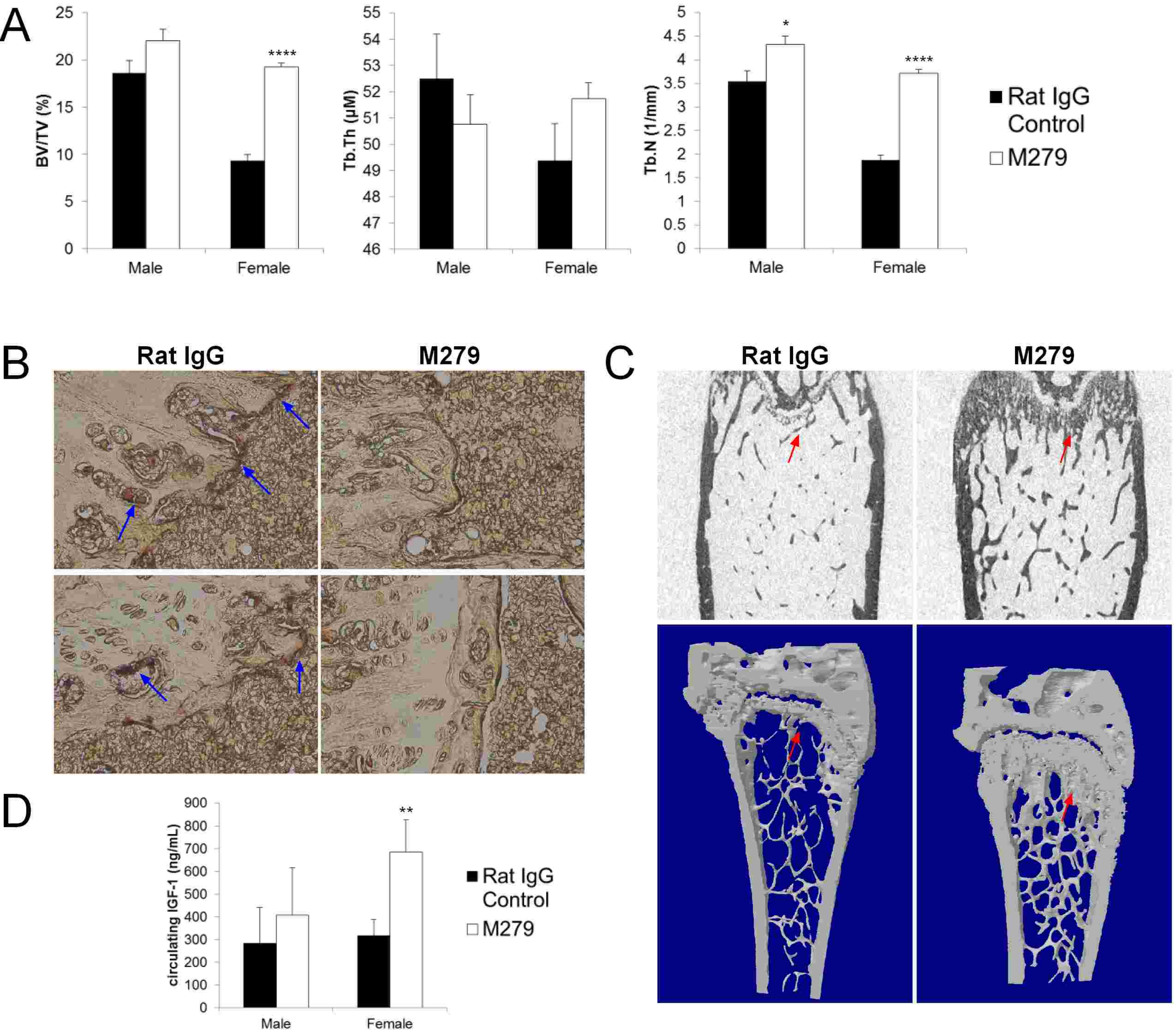
A
BV/TV (%)
Tb.Th (μM)
Tb.N (1/mm)
Male
Female
****
*
****
Rat IgG Control
M279
B
Rat IgG
M279
C
Rat IgG
M279
D
circulating IGF-1 (ng/mL)
**
Male
Female
Rat IgG Control
M279

FIGURE 4

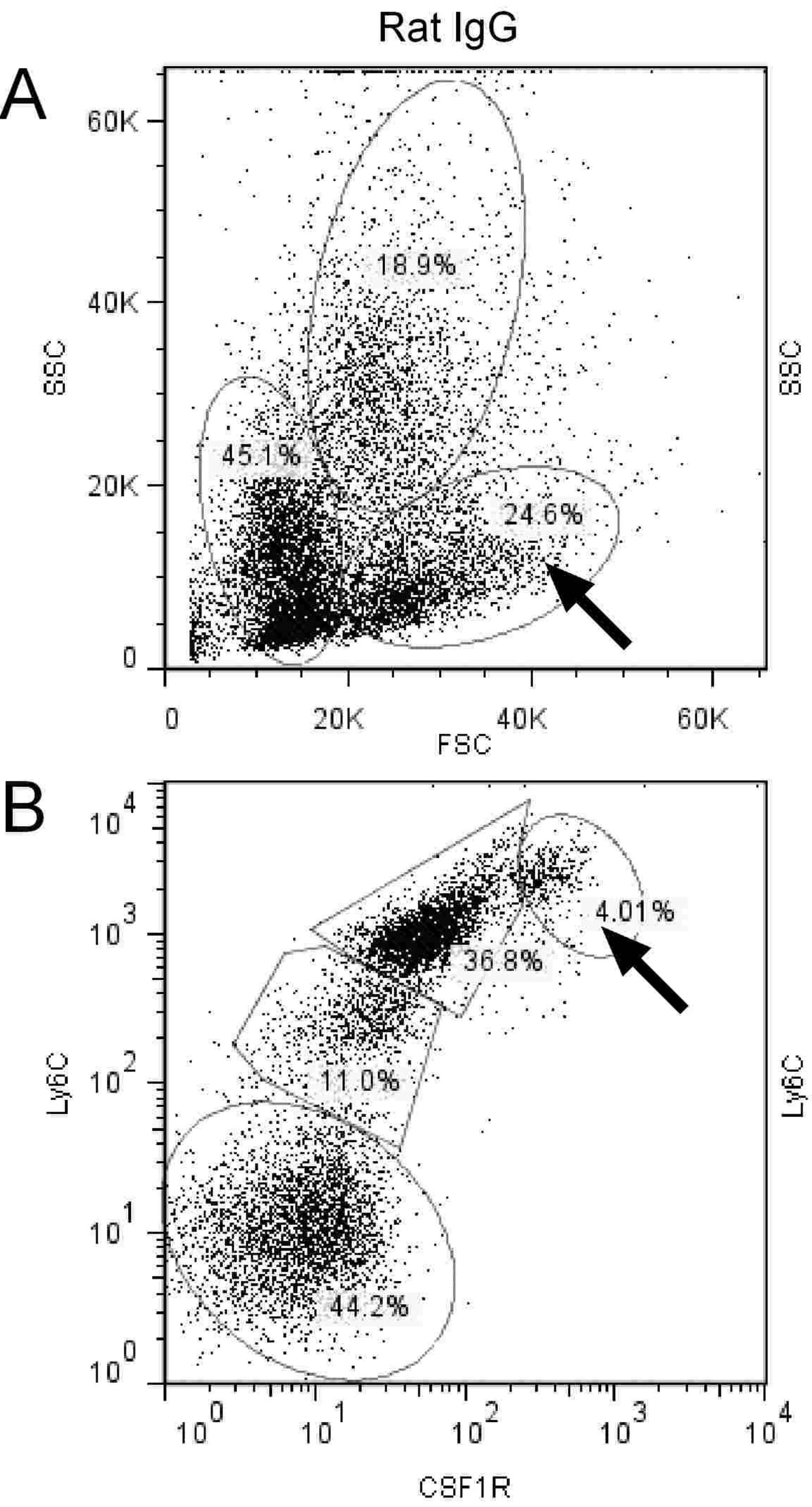
Rat IgG
M279
A
60K
40K
20K
0
SSC
18.9%
45.1%
24.6%
21.3%
42.9%
19.2%
0
20K
40K
60K
FSC
B
Ly6C
4.01%
36.8%
11.0%
44.2%
0.215%
46.4%
10.0%
41.7%
CSF1R

FIGURE 5

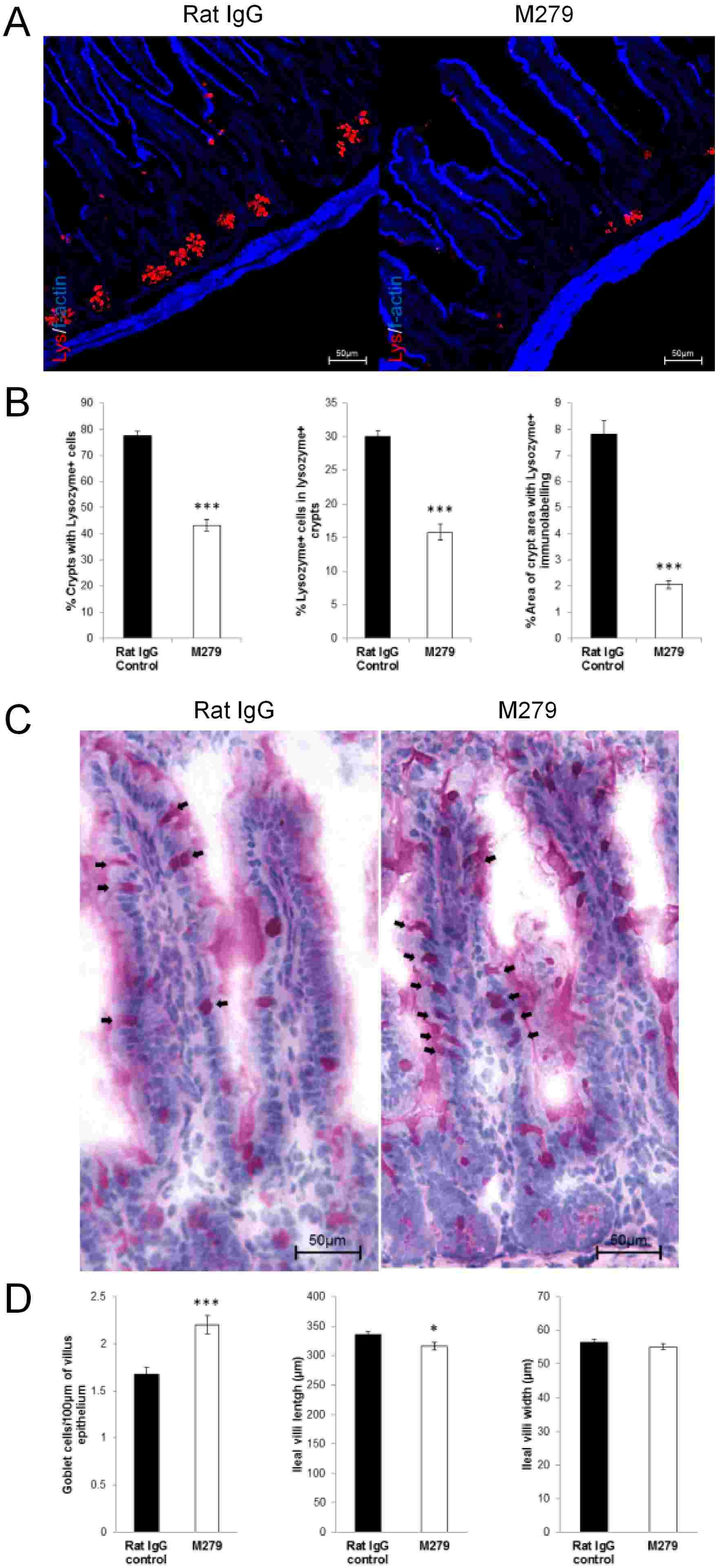
A
Rat IgG
M279
Lys/f-actin
50μm
B
% Crypts with Lysozyme+ cells
% Lysozyme+ cells in lysozyme+ crypts
% Area of crypt area with Lysozyme+ immunolabelling
Rat IgG Control
M279
***
C
Rat IgG
M279
50μm
D
Goblet cells/100μm of villus epithelium
Ileal villi length (μm)
Ileal villi width (μm)
Rat IgG control
M279
***
*

FIGURE 6

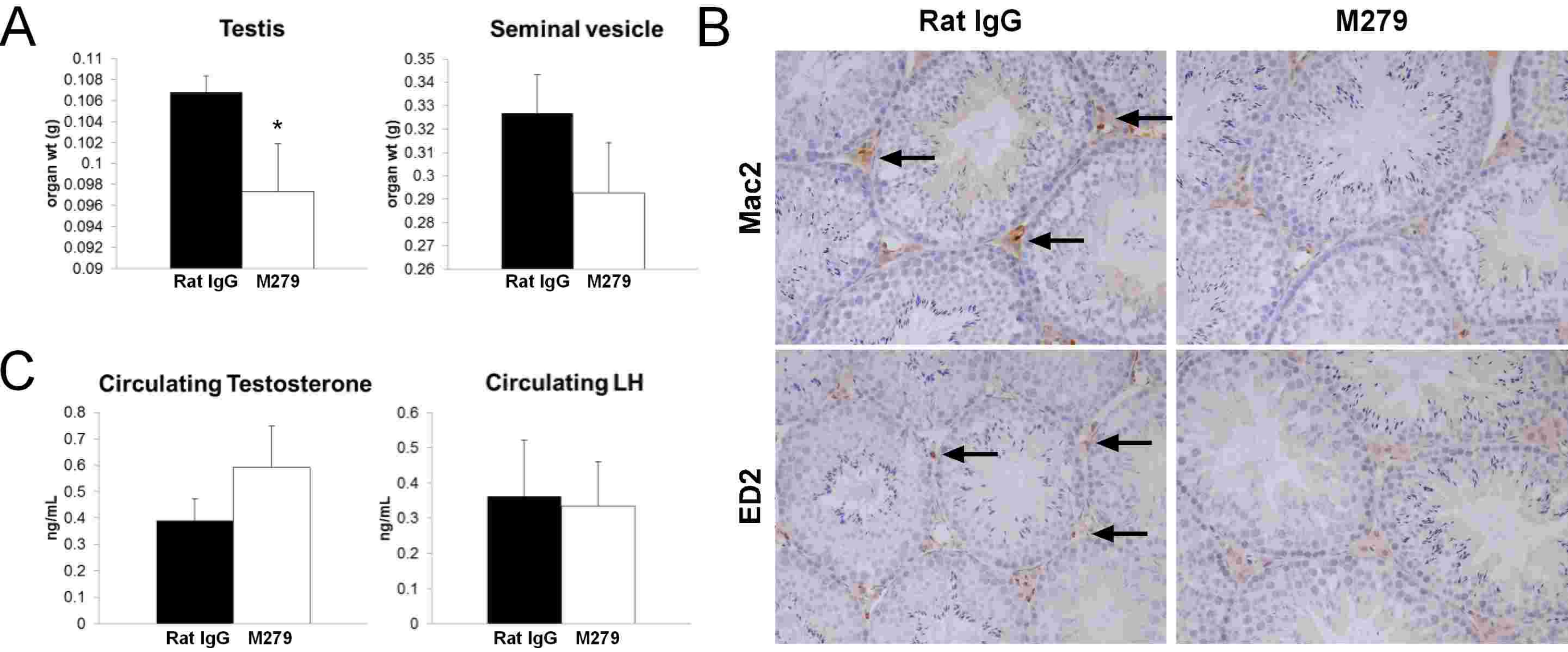

A
Testis
organ wt (g)
0.11
0.108
0.106
0.104
0.102
0.1
0.098
0.096
0.094
0.092
0.09
*
Rat IgG
M279
Seminal vesicle
organ wt (g)
0.35
0.34
0.33
0.32
0.31
0.3
0.29
0.28
0.27
0.26
Rat IgG
M279
B
Rat IgG
M279
Mac2
ED2
C
Circulating Testosterone
ng/mL
0.8
0.7
0.6
0.5
0.4
0.3
0.2
0.1
0
Rat IgG
M279
Circulating LH
ng/mL
0.6
0.5
0.4
0.3
0.2
0.1
0
Rat IgG
M279
