# Supplementary figures and images for: Pleiotropic effects of extended blockade of CSF1R signaling in adult mice
Source: J Leukoc Biol. 2014 Aug;96(2):265–74. doi: 10.1189/jlb.2A0114-006R (PMC4378363; doi:10.1189/jlb.2A0114-006R)

FIGURE S4

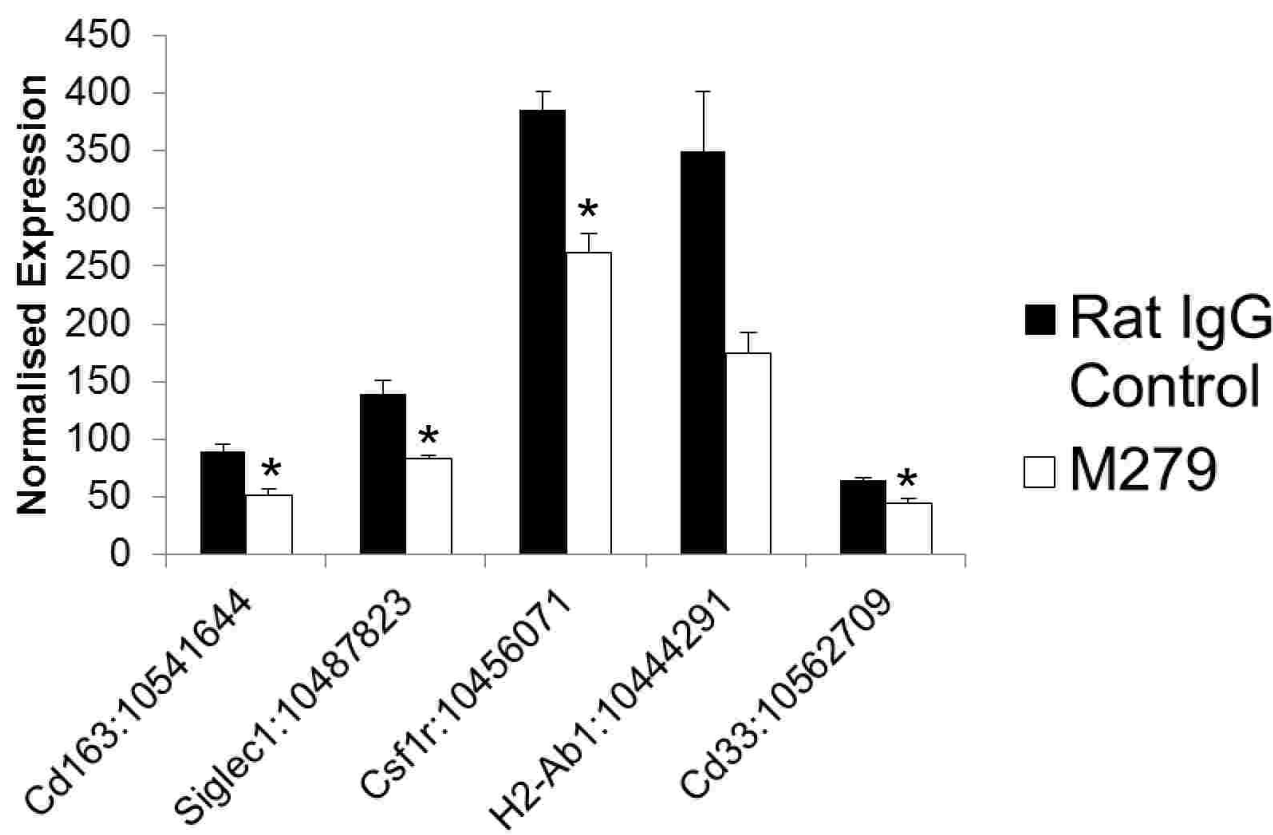

Supplement: Supplemental Data [file supp_jlb.2A0114-006R_jlb.2A0114-006RSuppData.zip › jlb.2A0114-006RSuppFig4.pdf]

FIGURE S2

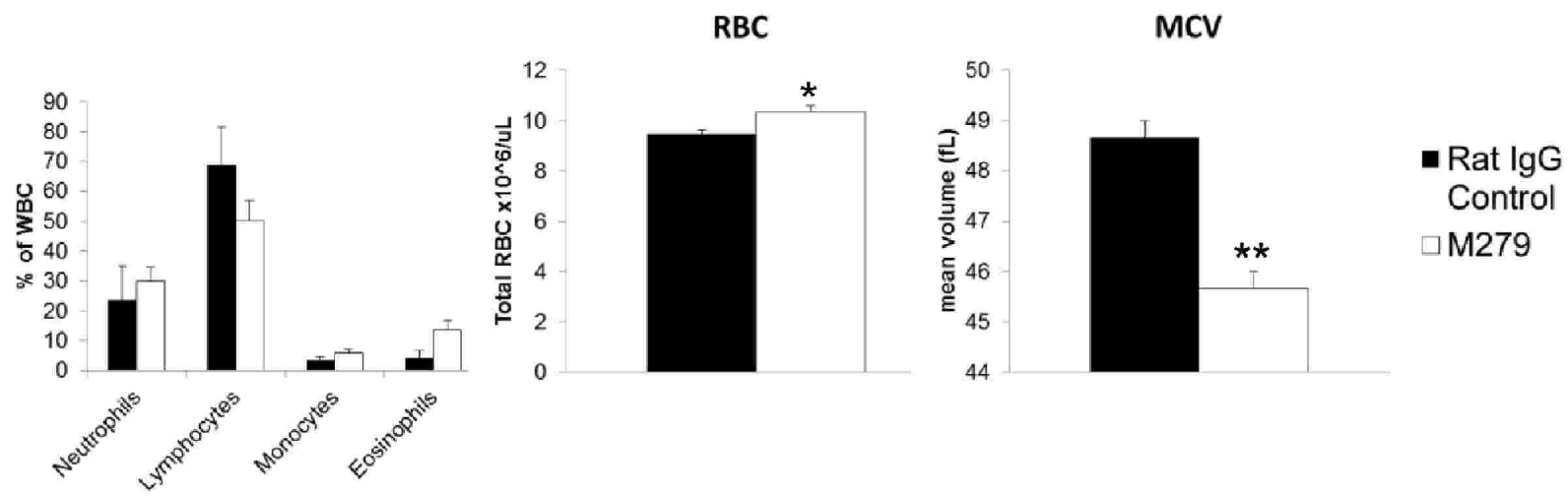

Supplement: Supplemental Data [file supp_jlb.2A0114-006R_jlb.2A0114-006RSuppData.zip › jlb.2A0114-006RSuppFig2.pdf]

FIGURE S3

Rat IgG Control

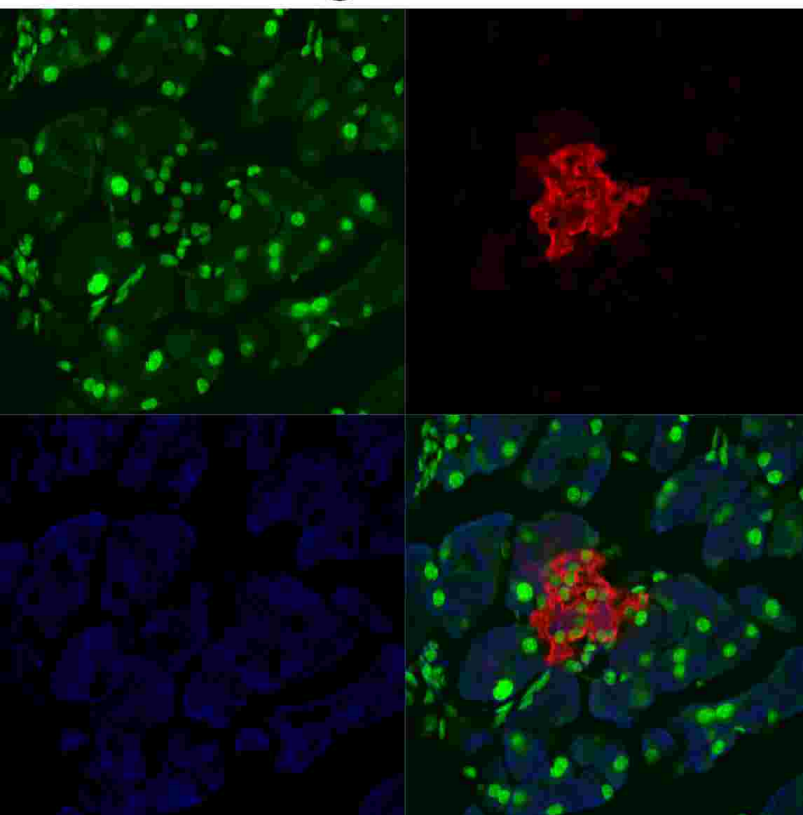

M279

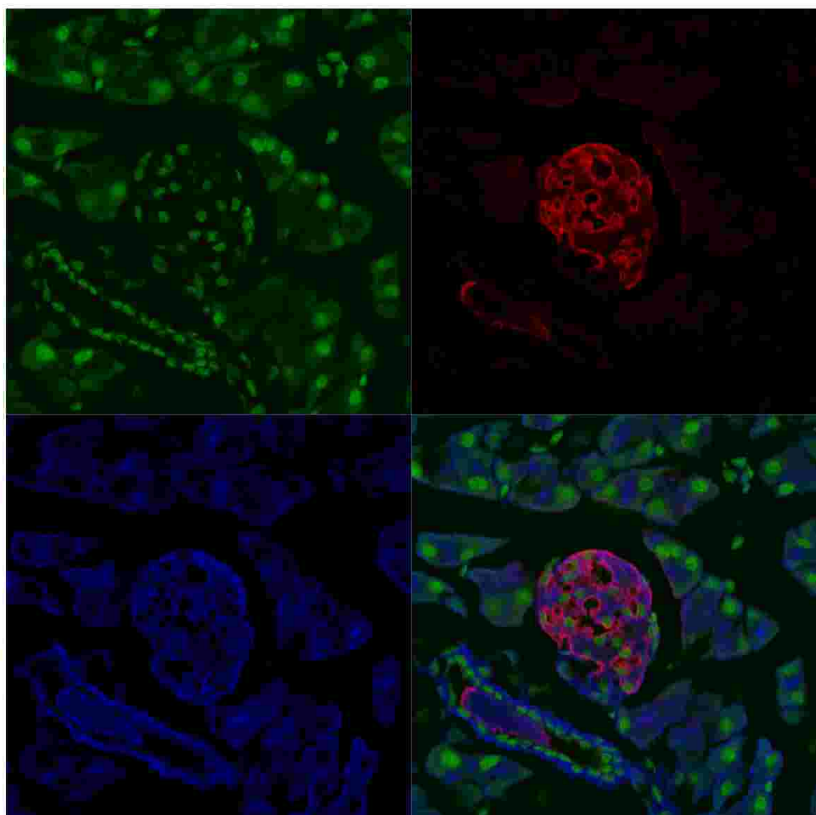

Supplement: Supplemental Data [file supp_jlb.2A0114-006R_jlb.2A0114-006RSuppData.zip › jlb.2A0114-006RSuppFig3.pdf]
